# Systematic Elucidation of the Mechanism of Quercetin against Gastric Cancer via Network Pharmacology Approach

**DOI:** 10.1155/2020/3860213

**Published:** 2020-09-03

**Authors:** Liangjun Yang, Zhipeng Hu, Jiajie Zhu, Qiting Liang, Hengli Zhou, Jiali Li, Xiangzhen Fan, Ziming Zhao, Huafeng Pan, Baoying Fei

**Affiliations:** ^1^Department of Gastroenterology, Tongde Hospital of Zhejiang Province, Hangzhou 310012, China; ^2^Hospital of Chengdu University of Traditional Chinese Medicine, Chengdu 610075, China; ^3^Science and Technology Innovation Center, Guangzhou University of Chinese Medicine, Guangzhou 510405, China; ^4^Guangdong Province Engineering Technology Research Institute of T.C.M., Guangzhou 510095, China

## Abstract

This study was aimed at elucidating the potential mechanisms of quercetin in the treatment of gastric cancer (GC). A network pharmacology approach was used to analyze the targets and pathways of quercetin in treating GC. The predicted targets of quercetin against GC were obtained through database mining, and the correlation of these targets with GC was analyzed by Gene Ontology (GO) and Kyoto Encyclopedia of Genes and Genomes (KEGG) pathway enrichment analyses. Next, the protein-protein interaction (PPI) network was constructed, and overall survival (OS) analysis of hub targets was performed using the Kaplan–Meier Plotter online tool. Finally, the mechanism was further analyzed via molecular docking of quercetin with the hub targets. Thirty-six quercetin-related genes were identified, 15 of which overlapped with GC-related targets. These targets were further mapped to 319 GO biological process terms and 10 remarkable pathways. In the PPI network analysis, six hub targets were identified, including AKT1, EGFR, SRC, IGF1R, PTK2, and KDR. The high expression of these targets was related to poor OS in GC patients. Molecular docking analysis confirmed that quercetin can bind to these hub targets. In conclusion, **t**his study provided a novel approach to reveal the therapeutic mechanisms of quercetin on GC, which will ease the future clinical application of quercetin in the treatment of GC.

## 1. Introduction

Gastric cancer (GC) is one of the most malignant cancers worldwide with poor prognosis. According to the global cancer statistics 2018, GC is the third common cause of cancer death, and it remains the most prevalent cancer in Eastern Asia, especially in China [1]. Although the advances in surgical methods, radiotherapy, chemotherapy, and neoadjuvant therapy have significantly improved the survival rates of patients with GC, the outlook for patients with advanced GC is still disappointing owing to poor prognosis and limited therapy options [2]. Thus, new treatments are needed.

Traditional Chinese medicine (TCM) is well known for its multitarget effects and fewer side effects, and it has huge advantages in improving the life quality of GC patients [3]. Natural products serve as a rich source of therapeutic compounds for treating diseases and developing synthetic drugs [4–6]. As a natural ingredient abundant in Chinese medicine, quercetin plays a vital role in antitumor therapy. A clinical study in Sweden has shown that dietary intake of quercetin can reduce the risk of GC, and the protective effect is particularly evident in women exposed to oxidative stress [7]. Experimental studies have shown that quercetin exerts antineoplastic function on GC cells by inhibiting proliferation and promoting apoptosis [8]. It can initiate autophagic progression in GC cells by modulating Akt-mTOR signaling and hypoxia-induced factor 1*α* (HIF-1*α*) signaling [9]. Moreover, quercetin has been proved to exert a gastroprotective effect owing to its antiperoxidative, antioxidant, and antihistaminic effects [10]. However, the mechanisms of quercetin against GC have not been comprehensively revealed.

Following the development of science and technology, a new analytical tool named network pharmacology has been applied in TCM research [11], and it has received much attention in recent years. As a brand-new area of pharmacology, network pharmacology provides new approaches for drug discovery for complex diseases and offers new methods for elucidating the multiple action mechanisms of drugs [12]. Mechanistically, the effects of quercetin are related to various targets and signaling pathways. To further explore the possible action mechanism of quercetin in the treatment of GC, network pharmacological analysis was performed to comprehensively investigate the potential mechanism. The predicted targets of quercetin against GC were obtained through database mining, and the correlation of these targets with GC was analyzed by Gene Ontology (GO) and Kyoto Encyclopedia of Genes and Genomes (KEGG) pathway enrichment analyses. Next, the protein-protein interaction (PPI) network was constructed, and overall survival (OS) analysis of hub targets was performed by the Kaplan–Meier Plotter online tool. Finally, the mechanism of quercetin was further analyzed via molecular docking. Thus, this study offered a powerful strategy for investigating the active mechanisms of quercetin in treating GC.

## 2. Materials and Methods

### 2.1. Drug-Likeness Prediction

Lipinski's rule of five (RO5) is a rule of thumb for screening potential oral drugs in humans by evaluating drug-likeness. The parameters include molecular weight (MW), XLogP3 (octanol-water partition coefficient), topological polar surface area, number of rotatable bonds, hydrogen bond acceptor count, and hydrogen bond donor count. To explore the drug-likeness properties of quercetin, the SMILES format (C1=CC(=C(C=C1C2=C(C(=O)C3=C(C=C(C=C3O2)O)O)O)O)O) of quercetin was uploaded into the SwissADME server (http://www.swissadme.ch), a web-based tool for evaluating pharmacokinetics, drug-likeness, and medicinal chemistry friendliness of small molecules [13]. Next, screening was performed under the default parameters.

### 2.2. Quercetin Target Prediction

The PharmMapper (http://lilab.ecust.edu.cn/pharmmapper/) and SwissTargetPrediction databases (http://www.swisstargetprediction.ch/) were used to predict the targets of quercetin. PharmMapper is a web server that identifies potential drug targets by matching the query compound to the internal pharmacophore model database via a reverse pharmacophore [14]. SwissTargetPrediction is a web server that predicts the targets of bioactive small molecules based on a combination of 2D and 3D similarity measures with known ligands [15]. The 3D molecular structure file and the canonical SMILES of quercetin were imported into the PharmMapper and SwissTargetPrediction databases, respectively. Next, the identified candidate targets were sent to the UniProt database (http://www.uniprot.org/) for normalization.

### 2.3. Collection of Targets Related to GC

To ensure a comprehensive collection of disease-related genes, GC-related genes were downloaded from four public database sources, including the Online Mendelian Inheritance in Man (OMIM) database (http://www.omim.org), MalaCards database (https://www.malacards.org/), Therapeutic Target Database (TTD, http://bidd.nus.edu.sg/group/cjttd/), and CooLGeN database (http://ci.smu.edu.cn/CooLGeN/). In the CooLGeN database, targets with hit scores higher than 5 were selected as GC-related genes [16]. Next, targets in the pathogenesis of GC were obtained.

### 2.4. KEGG and GO Enrichment

KEGG is a knowledge database famous for its pathway information, including graphical diagrams of biochemical pathways [17]. GO is a comprehensive source of functional genomics, and it includes the definitions of concepts related to gene functions [18]. To investigate the biological effects of quercetin, KEGG and GO enrichment analyses were conducted using the Comparative Toxicogenomics Database (CTD; http://ctdbase.org/), which is a robust, publicly available database integrated with functional and pathway data [19]. The subsequent pathways related to GC were selected based on pathological and clinical data.

### 2.5. Protein-Protein Interaction Analysis

PPI plays a significant role in biological processes and is vital for understanding the complex mechanisms in a living cell [20]. PPI network mapping was performed on the obtained bioactive ingredients and disease targets using the Search Tool for the Retrieval of Interacting Genes database (http://string-db.org/; version 10.5) with the species limited to “*Homo sapiens*” and a confidence score of >0.4. Next, a direct and an indirect target-target regulatory network graph of quercetin treatment against GC were obtained.

The PPI network was constructed by Cytoscape (version 3.6.1), a bioinformatics software used for data visualization and integration [21]. To find clusters (highly interconnected regions) within the PPI network, the Cytoscape plugin cytoHubba (version 0.1) was used. Top-ranked proteins were defined as hub targets based on the degree level.

### 2.6. Overall Survival Analysis of Hub Genes

To explore the effect of hub targets on the OS of GC patients, a cancer genomics dataset named the Kaplan–Meier Plotter (http://kmplot.com/analysis/index.php?p=service) [22] was used to estimate the prognostic significance of each hub gene. Patients with GC were divided into the high and low expression groups, and the two groups were compared by using a Kaplan–Meier survival plot. The hazard ratio (HR) with 95% confidence intervals (CI) and logrank *P* value were calculated.

### 2.7. Molecular Docking

To gain an in-depth insight into the relationship and action mechanisms between candidate proteins and quercetin, molecular docking was conducted to assess the strength and mode of interactions between quercetin and the hub targets. The molecular docking simulation was conducted by CB-Dock (http://cao.labshare.cn/cb-dock/), a new blind docking method based on cavity detection. It can automatically identify the binding sites of a given protein, calculate the center and size, customize the docking box size according to the query ligands, and then perform molecular docking with a popular docking program, AutoDock Vina [23]. The crystal structures of the hub targets were downloaded from the protein data bank (http://www.rcsb.org). The 3D structure of quercetin was downloaded from the PubChem compound database (https://pubchem.ncbi.nlm.nih.gov/). Next, the crystal structures of proteins and the ligand file of quercetin were inputted to CB-Dock, and docking analysis was conducted to elucidate the binding activities.

## 3. Results

### 3.1. Molecular Properties of Quercetin

Drug-likeness evaluation is vital for predicting potential therapeutic ligands. According to Lipinski's RO5, the MV of a drug-like compound should be less than 500 g/mol, the polar surface area (PSA) less than or equal to 140 A^2^, the calculated octanol/water partition coefficient (XLogP3) less than 5, the rotatable bond less than 10, the hydrogen bond acceptors no more than 10, and the hydrogen bond donors no more than 5 [16]. Our results showed that the properties of quercetin were in line with the RO5, indicating that it had good drug-like properties (Table 1).

### 3.2. Target Identification and Analysis

A drug usually binds to multiple targets, which is a characteristic known as polypharmacology or drug promiscuity [24]. Therefore, understanding the principles behind drug-target interactions is important for the treatment of diseases. In this study, the PharmMapper and SwissTargetPrediction databases were used to predict the targets of quercetin. After merging the target data, 36 duplicated targets for quercetin were saved (Table S1). Moreover, GC-related genes were retrieved from the OMIM, MalaCards, TTD, and CooLGeN databases. Finally, 990 target genes were obtained from the above databases after redundant data were deleted. Detailed information on these targets is described in Table S2.

**Figure 1:**
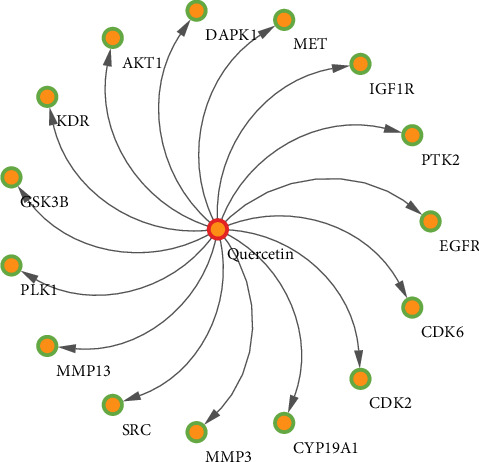
Compound-target network.

From an intersection of quercetin compound targets and GC targets, 15 overlapping genes were selected as targets of GC treatment. To understand the relationship between the targets and quercetin, a compound-target network (C-T network) was built. Quercetin, the targets, and the interactions between them, which had 16 nodes and 15 edges, are presented in Figure 1.

### 3.3. GO Enrichment Analysis

**Figure 2:**
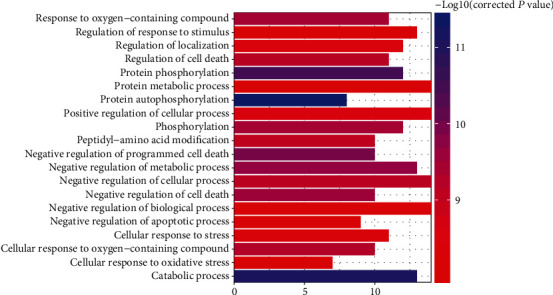
GO analysis of target genes.

To gain insights into the role of quercetin in various biological processes against GC, GO analysis was conducted for 15 targets. According to GO enrichment, these targets were significantly assigned to 319 GO biological process terms. The GO terms, corrected *P* value, and gene counts are provided in Table S3. As shown in Figure 2, the top 20 terms in biological processes were significantly related to “catabolic process” (GO:0009056), “negative regulation of programmed cell death” (GO:0043069), and “negative regulation of metabolic process” (GO:0009892). The results indicated that these targets not only modulated programmed cell death and cell proliferation but were also involved in phosphorylation, metabolic, and oxidative stress.

### 3.4. KEGG Enrichment Analysis

**Figure 3:**
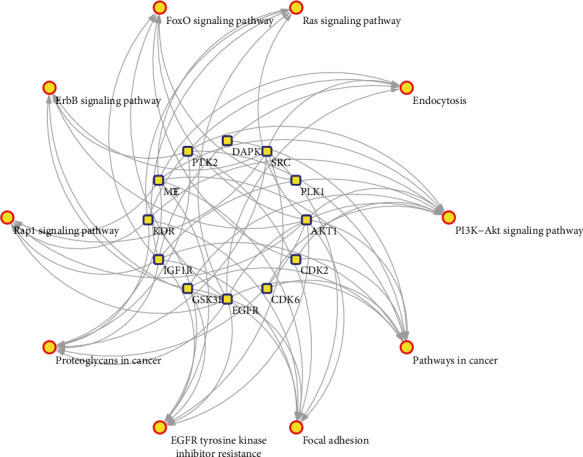
Target-pathway network.

To further uncover the potential pharmacological mechanisms of quercetin against GC, KEGG pathway analysis based on the CTD database was performed to determine the potential biological roles of these 15 genes. After pathway enrichment, 15 targets were mapped to 44 pathways. Combining the pathogenesis of gastric cancer and the gene count (gene count ≥ 5), pathways with no association with GC, such as melanoma (KEGG:hsa05218), prostate cancer (KEGG:hsa05215), and breast cancer pathways (KEGG:hsa05224), were deleted. Finally, 10 remarkably enriched pathways were shown as likely to be the major pathways in the treatment of GC (shown in Table 2). The target-pathway network was subsequently generated by mapping the targets to the major target pathways (Figure 3). The above results indicated that quercetin exerted its therapeutic effects by directly targeting GC-associated proteins and modulating the pathways involved in the pathological process.

### 3.5. Integration of Protein-Protein Interaction Network

**Figure 4:**
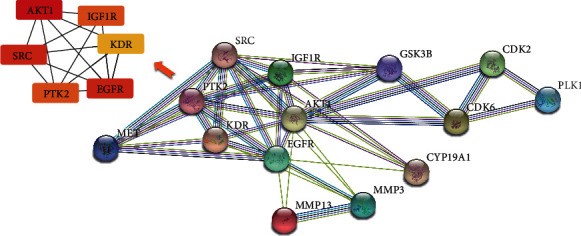
Protein-protein interaction network.

To visualize and quantify the function of proteins in cells at the systematic level, the PPI network of 15 targets for quercetin was constructed through the STRING database (Figure 3). According to the protein interaction network diagram, AKT1 was in the center of the network, showing the largest degree (degree = 11), followed by EGFR, SRC, IGF1R, PTK2, KDR, MMP3, MET, GSK3B, CYP19A1, CDK6, MMP13, CDK2, and PLK1. Based on the calculation of cytoHubba, six proteins including AKT1, EGFR, SRC, IGF1R, PTK2, and KDR with degrees > 5 were selected as hub targets that might play a critical role in the progression of GC. As shown in Figure 4, higher degree values are indicated by color changes from red to yellow.

### 3.6. Survival Analysis of Hub Genes

**Figure 5:**
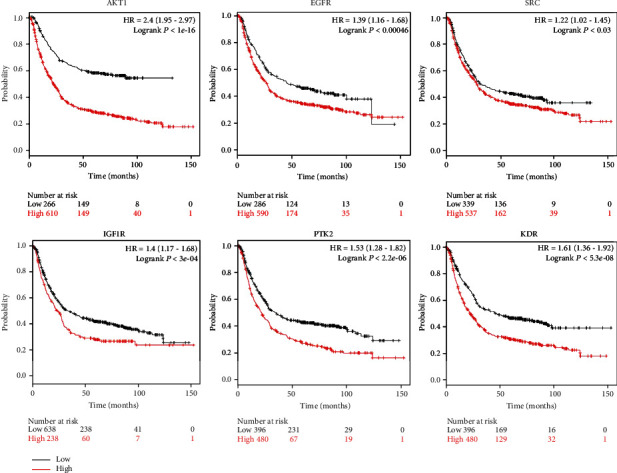
Prognostic value of the expression of the six hub genes. Survival data were analyzed by the Kaplan–Meier Plotter database (*P* < 0.05). Patients showing expression above the median are indicated by the red line, whereas the black line represents expression below the median. HR represents the hazard ratio.

Survival analysis on hub genes in GC patients was conducted based on the Kaplan–Meier Plotter database. The results showed that high mRNA expression of these hub genes, including AKT1, EGFR, SRC, IGF1R, PTK2, and KDR, was associated with poor OS in GC patients (Figure 5).

### 3.7. Confirmation of Hub Target by Molecular Docking

To verify the reliability of the drug-target interactions, the six hub proteins were selected as targets for molecular docking based on the network pharmacology results. The structure of quercetin was uploaded to CB-DOCK for analysis of docking potential with AKT1, EGFR, SRC, IGF1R, PTK2, and KDR. The Vina scores, cavity sizes, docking centers, and sizes of the predicted cavities from the docking simulation for each target protein are shown in Table 3. Vina score is usually regarded to represent the binding activity between protein and ligand. The more negative the Vina score, the more stable the binding of the compound to the target. Furthermore, if the size of the cavity is close to or larger than the compound, the accuracy of docking will increase [23, 25].

**Figure 6:**
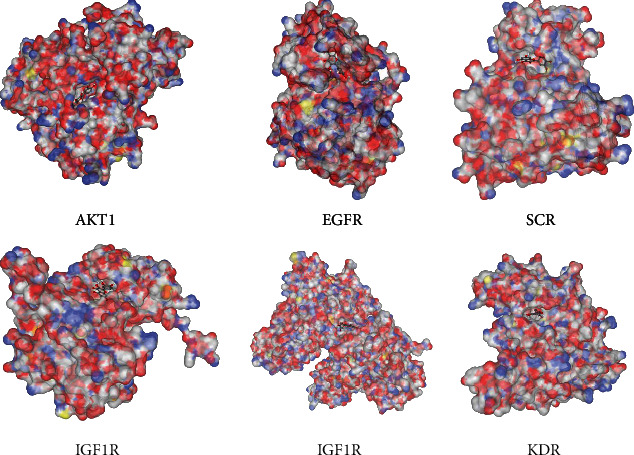
Docking results of quercetin and the six hub proteins.

The results showed that according to the Vina score and cavity size, there was a strong interaction between quercetin and the six hub proteins, which suggested the activity of quercetin in the treatment of GC. All docking sketch maps of the target protein with quercetin are shown in Figure 6.

The crystal structure of the protein active site is colored white (carbon), red (oxygen), blue (nitrogen), and yellow (sulfur). The crystal pose of the ligand is colored white (hydrogen), grey (carbon), and red (oxygen).

## 4. Discussion

Quercetin, a natural component from plants, has been reported to exert anti-GC activity via various mechanisms [9, 26]. The anticancer property of quercetin is becoming a research hotspot in recent years. The effect of quercetin against GC has been confirmed by experimental and clinical studies. However, the exact molecular mechanisms of quercetin against GC are still not completely understood. In this study, a network pharmacology approach integrating drug-likeness evaluation, target identification, GO and pathway analyses, and PPI analysis was successfully established to systematically analyze the potential molecular mechanism of quercetin in treating GC. According to the results of the RO5 parameters, quercetin had good drug-like properties, suggesting its potential as a chemotherapeutic agent against GC.

### 4.1. GO Biological Process Analysis

From the PharmMapper and SwissTargetPrediction databases, 15 targets were screened for GC and were shown to participate in 319 GO biological process terms that are mainly involved in cell apoptosis, proliferation, cell metabolic, and oxidative stress. It is well known that one of the most fundamental traits of cancer cells is the abnormal changes in proliferation [27] and apoptosis [28]. Thus, modulating the balance of proliferation and apoptosis in GC cells is an important way to treat GC. It has been revealed that quercetin can induce apoptotic cell death and antiproliferation in human GC cells by decreasing the antiapoptotic proteins Mcl-1, Bcl-2, and Bcl-x, but increasing the proapoptotic proteins Bad, Bax, and Bid [8]. With the emergence of metabolomics, the relationship between metabolic regulation and cancer has gained increasing attention. Disorders of metabolic reactions in the mitochondria often lead to the production of reactive species, which mainly include reactive oxygen species (ROS) [29]. Tumor cells, which exhibit hypermetabolism status, require high ROS concentrations to maintain their high proliferation rate [29]. Studies have shown that abnormal metabolism and oxidative stress are involved in the pathogenesis of GC [30–32]. Thus, modulation of metabolism and oxidative stress will have a positive impact on cancer therapy [33]. Studies have shown that quercetin has a wide range of biological actions, including modulating effect on metabolic homeostasis and oxidative stress [34, 35]. Furthermore, quercetin protects gastric epithelial cells by inhibiting oxidative stress and regulating mitochondrial dysfunction [36]. However, the effects of quercetin on cell metabolism and oxidative stress in QC remain unknown. Thus, further research is needed to uncover its potential function in GC therapy.

### 4.2. Pathway Analysis

KEGG pathway analysis was performed to better understand the mechanism of action of quercetin. The results suggested that quercetin could prevent GC through multiple pathways. Based on pathway analysis, the highly enriched pathways of quercetin in treating GC were associated with the PI3k-Akt signaling, EGFR tyrosine kinase inhibitor resistance, Rap1 signaling, ErbB signaling, FoxO signaling, and Ras signaling pathways. As a central regulatory pathway, the PI3K-Akt signaling pathway is frequently activated by genomic amplification in GC [37]. It plays a vital role in the regulation of tumorigenesis, including cell proliferation, cell viability, and cell apoptosis [38]. Some researchers have confirmed that quercetin induces mitochondrial-dependent apoptosis by inhibiting PI3K-Akt signaling, thereby inhibiting the growth of human GC stem cells [39]. The epidermal growth factor receptor (EGFR) is a member of the receptor tyrosine kinase ErbB family and is the expression product of prooncogene ErbB1. Abnormal expression of EGFR activates intracellular signaling cascades and controls vital cellular processes; thus, EGFR is a pivotal oncogene in GC progression [40, 41]. Cetuximab, an EGFR-targeted drug, has been used to treat GC by inducing EGFR internalization, downregulation, and degradation [42]. The resistance of EGFR tyrosine kinase inhibitor presents a challenge for the treatment of GC [43]. Evidence has proved that quercetin is effective in preventing cancer progression by inhibiting the EGFR signaling pathway and can reverse tamoxifen resistance in breast cancer cells [44, 45]. Therefore, it is of great significance to prove whether quercetin plays a therapeutic role by improving EGFR resistance in GC. FOXOs are transcription factors that orchestrate programs of gene expression known to play crucial roles in the cell biology of GC, including cell proliferation, apoptosis, differentiation, and metabolic responses [46, 47]. FOXOs are usually considered as tumor suppressors, and dysregulation of the functioning of FOXO proteins is associated with cancer progression and tumorigenesis [48]. Multiple pathways, such as the PI3K/AKT, Ras/MEK/ERK, and AMPK pathways, are associated with FOXOs in tumorigenesis [49]. Hence, targeting the FOXO signaling pathway could be an efficient way for the discovery and development of efficacious agents against cancer [50]. A previous study has shown that quercetin can suppress tumor growth with the induction of FOXO1 activation [51]. However, whether quercetin can treat GC by activating the FoxO signaling pathway still needs further validation. As a small GTPase in the Ras-related protein family, Ras-associated protein-1 (Rap1) plays a vital role in the regulation of cell migration, invasion, and metastasis. Many studies have implicated that Rap1 activity is increased in various cancers [52, 53]. Although the sequences of Ras and Rap are similar, their activators and effector pathways are different. Thus, Rap1 is considered to function as an antagonist of Ras signaling [54]. In the present study, quercetin was shown to affect the Ras and Rap1 signaling pathways. This is consistent with a previous finding that quercetin downregulates the levels of oncogenic Ras in cancer cells [55]. Nevertheless, the relationship between quercetin and Rap1 is still unknown, which provides a new approach for revealing the mechanism of quercetin in treating GC. Taken together, the results showed that quercetin can treat GC through multiple pathways, and the specific process by which quercetin affects these pathways requires further exploration.

### 4.3. Hub Target Analysis

To elucidate the significance of quercetin targets, a PPI network was constructed. Based on the network, the top six hub genes were identified, namely, AKT1, EGFR, SRC, IGF1R, PTK2, and KDR. Next, survival analysis showed that high expression of these genes was related to poor OS in GC patients. To investigate the mechanism of the interactions between quercetin and the six hub targets, molecular docking was employed. The results indicated that quercetin can bind with the large binding site of AKT1, EGFR, SRC, IGF1R, PTK2, and KDR with good binding scores, which showed better affinity with quercetin. The protooncogene Akt, which comprised Akt1, Akt2, and Akt3, is a well-characterized serine/threonine kinase that lies downstream of PI3K. In GC pathogenesis, activated Akt1 induces cell proliferation, survival, and metastasis by regulating the activity of several downstream molecules [56, 57]. Several studies have proven that quercetin can attenuate cancer cell migration and invasion by suppressing the protein levels of Akt1 [58, 59], which is consistent with the predicted result. EGFR, a member of the ErbB family receptors, is well known for its dominant role in tumorigenesis and development. It is closely related to gastric mucosa proliferation in GC and is associated with poor prognosis in GC [60]. Clinical studies have shown that overall response rates under treatment with cetuximab, an EGFR-targeting monoclonal antibody, vary from 40 to 60% [61, 62], which suggested its therapeutic effects in GC patients. Src, a serine/threonine kinase, is important in the development of many solid tumors [63]. Compared with that in the normal tissues, Src is commonly overexpressed or activated during GC development [64, 65]. Consequently, activated Src can regulate cell proliferation, angiogenesis, invasion, and metastasis by transducing the PI3K, Ras, and MYC pathways [66]. IGF1R is a transmembrane receptor tyrosine kinase that promotes the progression and metastatic ability of GC [67]. It can inhibit the apoptosis of cancer cells through the activation of SRC and PI3K/AKT [68, 69]. As a cytoplasmic protein tyrosine kinase, PTK2 can enhance cell motility, survival, and proliferation through effects on cancer cells and stromal cells in the tumor microenvironment [70]. PTK2 inhibitors are able to decrease tumor growth, metastasis, and angiogenesis and thus can act as promising chemotherapeutics [71, 72]. KDR, also known as VEGF receptor-2, is a key receptor involved in malignant angiogenesis. High KDR expression is one of the characteristics of GC; therefore, KDE is considered a potential therapeutic target in GC treatment [73, 74]. It has been shown that ramucirumab, which specifically targets KDR, has survival benefits in patients with advanced GC [75], which suggested its role as a therapeutic agent. Taken together, AKT1, EGFR, SRC, IGF1R, PTK2, and KDR are crucial in the pathogenesis of GC. These targets may be the key points of the therapeutic action of quercetin in GC, although several experiments have proven that quercetin can inhibit the expression of AKT1 [76], EGFR [77], SRC [78], IGF1R [79], PTK2 [80], and KDR [81]. Thus, the exact mechanism of quercetin on GC based on molecular docking requires further validation in biological experiments.

## 5. Conclusions

This study provided a novel approach to reveal the therapeutic mechanisms of quercetin against GC. The results showed that quercetin may exert an anti-GC effect through multiple targets, pathways, and biological processes. However, further studies are required to confirm the clinical efficacy of quercetin and its mechanisms against GC.

## Figures and Tables

**Table 1:** Molecular properties of quercetin.

Property	Value
Molecular weight	302.24 g/mol
PSA	131.36 A^2^
XLogP3	1.54
Rotatable bonds	1
H-bond donor	5
H-bond acceptor	7
Molar refractivity	78.03
Bioavailability score	0.55

**Table 2:** Top 10 representative pathways according to gene count.

Pathway ID	Pathway	Corrected *P* value	Gene count	Annotated genes
KEGG:hsa04151	PI3K-Akt signaling pathway	2.34*E*-13	9	AKT1, CDK2, CDK6, EGFR, GSK3B, IGF1R, KDR, MET, PTK2
KEGG:hsa05200	Pathways in cancer	8.86*E*-13	9	AKT1, CDK2, CDK6, DAPK1, EGFR, GSK3B, IGF1R, MET, PTK2
KEGG:hsa04510	Focal adhesion	5.02*E*-13	8	AKT1, EGFR, GSK3B, IGF1R, KDR, MET, PTK2, SRC
KEGG:hsa01521	EGFR tyrosine kinase inhibitor resistance	1.51*E*-13	7	AKT1, EGFR, GSK3B, IGF1R, KDR, MET, SRC
KEGG:hsa05205	Proteoglycans in cancer	1.30*E*-10	7	AKT1, EGFR, IGF1R, KDR, MET, PTK2, SRC
KEGG:hsa04015	Rap1 signaling pathway	2.71*E*-08	6	AKT1, EGFR, IGF1R, KDR, MET, SRC
KEGG:hsa04012	ErbB signaling pathway	3.76*E*-08	5	AKT1, EGFR, GSK3B, PTK2, SRC
KEGG:hsa04068	FoxO signaling pathway	3.31*E*-07	5	AKT1, CDK2, EGFR, IGF1R, PLK1
KEGG:hsa04014	Ras signaling pathway	5.04*E*-06	5	AKT1, EGFR, IGF1R, KDR, MET
KEGG:hsa04144	Endocytosis	9.94*E*-06	5	EGFR, IGF1R, KDR, MET, SRC

**Table 3:** Vina scores and cavity information of the docking simulation pose for each targeted protein and quercetin.

Receptors	PDB ID	Vina score	Cavity size	Center			Size		
				*x*	*y*	*z*	*x*	*y*	*z*
AKT1	6s9x	-9.7	2347	2	8	13	32	27	35
EGFR	6s9b	-7.9	1392	-54	30	-1	35	35	21
SCR	6ate	-10.1	717	-2	-34	9	27	21	21
IGF1R	5fxr	-8.1	844	17	-6	52	27	21	21
PTK2	2aeh	-8.5	2041	8	122	22	31	29	31
KDR	6gqo	-9.4	1084	17	2	9	21	21	21

## Data Availability

The data used to support the findings of this study are available from the corresponding authors upon request.

## Supplementary Materials

Supplementary materials Table S1: information on quercetin-related targets. Table S2: information on GC-related targets. Table S3: GO terms of therapy target genes and their corresponding count, corrected *P* values, and gene count.
