# Supplementary material for: Systematic Elucidation of the Mechanism of Quercetin against Gastric Cancer via Network Pharmacology Approach
Source: Biomed Res Int. 2020 Sep 3;2020:3860213. doi: 10.1155/2020/3860213 (PMC7486643; doi:10.1155/2020/3860213)
Supplement: Supplementary materials — Table S1: information on quercetin-related targets. Table S2: information on GC-related targets. Table S3: GO terms of therapy target genes and their corresponding count, corrected P values, and gene count. [file 3860213.f1.docx]

**Table S1**

The information of quercetin-related targets.

| **No.** | **Targets** | **Organism** |
| --- | --- | --- |
| 1 | EGFR | Homo sapiens |
| 2 | CDK6 | Homo sapiens |
| 3 | CDK2 | Homo sapiens |
| 4 | CYP19A1 | Homo sapiens |
| 5 | MMP3 | Homo sapiens |
| 6 | SRC | Homo sapiens |
| 7 | MMP13 | Homo sapiens |
| 8 | PLK1 | Homo sapiens |
| 9 | GSK3B | Homo sapiens |
| 10 | KDR | Homo sapiens |
| 11 | AKT1 | Homo sapiens |
| 12 | DAPK1 | Homo sapiens |
| 13 | MET | Homo sapiens |
| 14 | IGF1R | Homo sapiens |
| 15 | PTK2 | Homo sapiens |
| 16 | CA2 | Homo sapiens |
| 17 | F2 | Homo sapiens |
| 18 | PIM1 | Homo sapiens |
| 19 | CA12 | Homo sapiens |
| 20 | TTR | Homo sapiens |
| 21 | BACE1 | Homo sapiens |
| 22 | ESR2 | Homo sapiens |
| 23 | CDK5R1 | Homo sapiens |
| 24 | PYGL | Homo sapiens |
| 25 | HSD17B1 | Homo sapiens |
| 26 | AKR1B1 | Homo sapiens |
| 27 | AKR1C1 | Homo sapiens |
| 28 | MMP12 | Homo sapiens |
| 29 | AKR1C3 | Homo sapiens |
| 30 | AKR1C2 | Homo sapiens |
| 31 | CSNK2A1 | Homo sapiens |
| 32 | SYK | Homo sapiens |
| 33 | INSR | Homo sapiens |
| 34 | ARG1 | Homo sapiens |
| 35 | PIK3R1 | Homo sapiens |
| 36 | GLO1 | Homo sapiens |

**Table S2**

The information of GC-related targets.

| **No.** | **Targets** | **Organism** |
| --- | --- | --- |
| 1 | A2M-AS1 | Homo sapiens |
| 2 | ABCB1 | Homo sapiens |
| 3 | ABHD11-AS1 | Homo sapiens |
| 4 | AFAP1-AS1 | Homo sapiens |
| 5 | AFDN-DT | Homo sapiens |
| 6 | AFP | Homo sapiens |
| 7 | AGAP2-AS1 | Homo sapiens |
| 8 | AKR7L | Homo sapiens |
| 9 | AKT1 | Homo sapiens |
| 10 | ANXA2P2 | Homo sapiens |
| 11 | APC | Homo sapiens |
| 12 | AURKA | Homo sapiens |
| 13 | BANCR | Homo sapiens |
| 14 | BAX | Homo sapiens |
| 15 | BCAR4 | Homo sapiens |
| 16 | BCL2 | Homo sapiens |
| 17 | BCL2L1 | Homo sapiens |
| 18 | BLACAT1 | Homo sapiens |
| 19 | BNC2-AS1 | Homo sapiens |
| 20 | CACYBP | Homo sapiens |
| 21 | CAPN9 | Homo sapiens |
| 22 | CASC15 | Homo sapiens |
| 23 | CASC2 | Homo sapiens |
| 24 | CASC9 | Homo sapiens |
| 25 | CASP10 | Homo sapiens |
| 26 | CASP3 | Homo sapiens |
| 27 | CBR3-AS1 | Homo sapiens |
| 28 | CCAT1 | Homo sapiens |
| 29 | CCAT2 | Homo sapiens |
| 30 | CCDC136 | Homo sapiens |
| 31 | CCKBR | Homo sapiens |
| 32 | CCND1 | Homo sapiens |
| 33 | CD44 | Homo sapiens |
| 34 | CDH1 | Homo sapiens |
| 35 | CDH17 | Homo sapiens |
| 36 | CDK2 | Homo sapiens |
| 37 | CDKN1A | Homo sapiens |
| 38 | CDKN1B | Homo sapiens |
| 39 | CDKN2A | Homo sapiens |
| 40 | CDKN2B | Homo sapiens |
| 41 | CDKN2B-AS1 | Homo sapiens |
| 42 | CDX1 | Homo sapiens |
| 43 | CDX2 | Homo sapiens |
| 44 | CEACAM5 | Homo sapiens |
| 45 | CEBPA-DT | Homo sapiens |
| 46 | CLDN4 | Homo sapiens |
| 47 | CNDP2 | Homo sapiens |
| 48 | CRNDE | Homo sapiens |
| 49 | CTNNA1 | Homo sapiens |
| 50 | CTNNB1 | Homo sapiens |
| 51 | CYTOR | Homo sapiens |
| 52 | DANCR | Homo sapiens |
| 53 | DLEU1 | Homo sapiens |
| 54 | DMTF1 | Homo sapiens |
| 55 | DNMT1 | Homo sapiens |
| 56 | DPYD | Homo sapiens |
| 57 | DUXAP8 | Homo sapiens |
| 58 | DUXAP9 | Homo sapiens |
| 59 | EGF | Homo sapiens |
| 60 | EGFLAM-AS1 | Homo sapiens |
| 61 | EGFR | Homo sapiens |
| 62 | EGOT | Homo sapiens |
| 63 | ENSG00000224220 | Homo sapiens |
| 64 | ENSG00000225032 | Homo sapiens |
| 65 | ENSG00000229717 | Homo sapiens |
| 66 | ENSG00000232406 | Homo sapiens |
| 67 | ENSG00000249201 | Homo sapiens |
| 68 | ENSG00000250406 | Homo sapiens |
| 69 | ENSG00000253389 | Homo sapiens |
| 70 | ENSG00000266990 | Homo sapiens |
| 71 | ENSG00000277200 | Homo sapiens |
| 72 | ENSG00000278769 | Homo sapiens |
| 73 | ENSG00000285159 | Homo sapiens |
| 74 | ENTPD1-AS1 | Homo sapiens |
| 75 | ERBB2 | Homo sapiens |
| 76 | ERVH48-1 | Homo sapiens |
| 77 | FENDRR | Homo sapiens |
| 78 | FER1L4 | Homo sapiens |
| 79 | FEZF1-AS1 | Homo sapiens |
| 80 | FGF7 | Homo sapiens |
| 81 | FGFR2 | Homo sapiens |
| 82 | FHIT | Homo sapiens |
| 83 | FLT4 | Homo sapiens |
| 84 | FRGCA | Homo sapiens |
| 85 | GACAT2 | Homo sapiens |
| 86 | GACAT3 | Homo sapiens |
| 87 | GAPLINC | Homo sapiens |
| 88 | GAS5 | Homo sapiens |
| 89 | GAST | Homo sapiens |
| 90 | GATA6-AS1 | Homo sapiens |
| 91 | GHET1 | Homo sapiens |
| 92 | GKN1 | Homo sapiens |
| 93 | GKN2 | Homo sapiens |
| 94 | GPR65 | Homo sapiens |
| 95 | GSTM1 | Homo sapiens |
| 96 | GSTP1 | Homo sapiens |
| 97 | H19 | Homo sapiens |
| 98 | HAGLR | Homo sapiens |
| 99 | HAGLROS | Homo sapiens |
| 100 | HGF | Homo sapiens |
| 101 | HIF1A | Homo sapiens |
| 102 | HIF1A-AS2 | Homo sapiens |
| 103 | HNF1A-AS1 | Homo sapiens |
| 104 | HOTAIR | Homo sapiens |
| 105 | HOTTIP | Homo sapiens |
| 106 | HOXA11-AS | Homo sapiens |
| 107 | HOXA13 | Homo sapiens |
| 108 | HOXA-AS2 | Homo sapiens |
| 109 | HULC | Homo sapiens |
| 110 | IL1B | Homo sapiens |
| 111 | IL1RN | Homo sapiens |
| 112 | INHBA-AS1 | Homo sapiens |
| 113 | INTS7 | Homo sapiens |
| 114 | IQGAP1 | Homo sapiens |
| 115 | IRF1 | Homo sapiens |
| 116 | JUN | Homo sapiens |
| 117 | KCNQ1OT1 | Homo sapiens |
| 118 | KLF6 | Homo sapiens |
| 119 | KRAS | Homo sapiens |
| 120 | KRT18P55 | Homo sapiens |
| 121 | KRT20 | Homo sapiens |
| 122 | KRT7-AS | Homo sapiens |
| 123 | LINC00052 | Homo sapiens |
| 124 | LINC00261 | Homo sapiens |
| 125 | LINC00572 | Homo sapiens |
| 126 | LINC00628 | Homo sapiens |
| 127 | LINC00668 | Homo sapiens |
| 128 | LINC00673 | Homo sapiens |
| 129 | LINC00901 | Homo sapiens |
| 130 | LINC00941 | Homo sapiens |
| 131 | LINC01006 | Homo sapiens |
| 132 | LINC01097 | Homo sapiens |
| 133 | LINC01234 | Homo sapiens |
| 134 | LINC01612 | Homo sapiens |
| 135 | LINC01772 | Homo sapiens |
| 136 | LINC01856 | Homo sapiens |
| 137 | LINC02461 | Homo sapiens |
| 138 | LINC-ROR | Homo sapiens |
| 139 | LNCRNA-ATB | Homo sapiens |
| 140 | LOC101929759 | Homo sapiens |
| 141 | LOC105372446 | Homo sapiens |
| 142 | MACC1-AS1 | Homo sapiens |
| 143 | MAGEA1 | Homo sapiens |
| 144 | MALAT1 | Homo sapiens |
| 145 | MAP3K20-AS1 | Homo sapiens |
| 146 | MAPK1 | Homo sapiens |
| 147 | MAPK3 | Homo sapiens |
| 148 | MDC1-AS1 | Homo sapiens |
| 149 | MEG3 | Homo sapiens |
| 150 | MET | Homo sapiens |
| 151 | MGMT | Homo sapiens |
| 152 | MIAT | Homo sapiens |
| 153 | MIR106A | Homo sapiens |
| 154 | MIR106B | Homo sapiens |
| 155 | MIR107 | Homo sapiens |
| 156 | MIR122 | Homo sapiens |
| 157 | MIR141 | Homo sapiens |
| 158 | MIR143 | Homo sapiens |
| 159 | MIR145 | Homo sapiens |
| 160 | MIR150 | Homo sapiens |
| 161 | MIR15B | Homo sapiens |
| 162 | MIR191 | Homo sapiens |
| 163 | MIR192 | Homo sapiens |
| 164 | MIR196B | Homo sapiens |
| 165 | MIR200C | Homo sapiens |
| 166 | MIR203A | Homo sapiens |
| 167 | MIR20A | Homo sapiens |
| 168 | MIR21 | Homo sapiens |
| 169 | MIR214 | Homo sapiens |
| 170 | MIR215 | Homo sapiens |
| 171 | MIR221 | Homo sapiens |
| 172 | MIR222 | Homo sapiens |
| 173 | MIR223 | Homo sapiens |
| 174 | MIR24-1 | Homo sapiens |
| 175 | MIR24-2 | Homo sapiens |
| 176 | MIR25 | Homo sapiens |
| 177 | MIR27A | Homo sapiens |
| 178 | MIR31HG | Homo sapiens |
| 179 | MIR34A | Homo sapiens |
| 180 | MIR423 | Homo sapiens |
| 181 | MIR4435-2HG | Homo sapiens |
| 182 | MIR451A | Homo sapiens |
| 183 | MIR512-1 | Homo sapiens |
| 184 | MIR92A2 | Homo sapiens |
| 185 | MIR93 | Homo sapiens |
| 186 | MLH1 | Homo sapiens |
| 187 | MMP2 | Homo sapiens |
| 188 | MMP7 | Homo sapiens |
| 189 | MMP9 | Homo sapiens |
| 190 | MSH2 | Homo sapiens |
| 191 | MSTO2P | Homo sapiens |
| 192 | MT1DP | Homo sapiens |
| 193 | MTHFR | Homo sapiens |
| 194 | MUC1 | Homo sapiens |
| 195 | MUC2 | Homo sapiens |
| 196 | MUC5AC | Homo sapiens |
| 197 | MUC6 | Homo sapiens |
| 198 | MUTYH | Homo sapiens |
| 199 | MYC | Homo sapiens |
| 200 | MYLK-AS1 | Homo sapiens |
| 201 | NCRUPAR | Homo sapiens |
| 202 | NEAT1 | Homo sapiens |
| 203 | NME1 | Homo sapiens |
| 204 | NPTN-IT1 | Homo sapiens |
| 205 | OR3A4P | Homo sapiens |
| 206 | PAK1 | Homo sapiens |
| 207 | PANDAR | Homo sapiens |
| 208 | PANTR1 | Homo sapiens |
| 209 | PCAT1 | Homo sapiens |
| 210 | PCNA-AS1 | Homo sapiens |
| 211 | PIK3CA | Homo sapiens |
| 212 | PLAU | Homo sapiens |
| 213 | PRDM16-DT | Homo sapiens |
| 214 | PRNCR1 | Homo sapiens |
| 215 | PRSS21 | Homo sapiens |
| 216 | PSCA | Homo sapiens |
| 217 | PTEN | Homo sapiens |
| 218 | PTENP1 | Homo sapiens |
| 219 | PTGS2 | Homo sapiens |
| 220 | PVT1 | Homo sapiens |
| 221 | PWRN1 | Homo sapiens |
| 222 | RARB | Homo sapiens |
| 223 | REG4 | Homo sapiens |
| 224 | RIOX2 | Homo sapiens |
| 225 | RMRP | Homo sapiens |
| 226 | RN7SK | Homo sapiens |
| 227 | RPL34-AS1 | Homo sapiens |
| 228 | RUFY3 | Homo sapiens |
| 229 | RUNX1-IT1 | Homo sapiens |
| 230 | RUNX3 | Homo sapiens |
| 231 | S100A8 | Homo sapiens |
| 232 | SERPINA3 | Homo sapiens |
| 233 | SERPINB5 | Homo sapiens |
| 234 | SFTA1P | Homo sapiens |
| 235 | SGK1 | Homo sapiens |
| 236 | SH3RF3-AS1 | Homo sapiens |
| 237 | SLC5A4-AS1 | Homo sapiens |
| 238 | SLC7A11-AS1 | Homo sapiens |
| 239 | SMAD2 | Homo sapiens |
| 240 | SMAD4 | Homo sapiens |
| 241 | SMIM31 | Homo sapiens |
| 242 | SNHG1 | Homo sapiens |
| 243 | SNHG12 | Homo sapiens |
| 244 | SNHG14 | Homo sapiens |
| 245 | SNHG15 | Homo sapiens |
| 246 | SNHG16 | Homo sapiens |
| 247 | SNHG20 | Homo sapiens |
| 248 | SNHG5 | Homo sapiens |
| 249 | SNHG6 | Homo sapiens |
| 250 | SNHG7 | Homo sapiens |
| 251 | SNHG8 | Homo sapiens |
| 252 | SOX2-OT | Homo sapiens |
| 253 | SPRY4-IT1 | Homo sapiens |
| 254 | SUCLG2-AS1 | Homo sapiens |
| 255 | SUMO1P3 | Homo sapiens |
| 256 | TACC1 | Homo sapiens |
| 257 | TCONS_00068220 | Homo sapiens |
| 258 | TDRG1 | Homo sapiens |
| 259 | TERT | Homo sapiens |
| 260 | TFF1 | Homo sapiens |
| 261 | TFF2 | Homo sapiens |
| 262 | TFF3 | Homo sapiens |
| 263 | TGFA | Homo sapiens |
| 264 | TGFB1 | Homo sapiens |
| 265 | TGFBR1 | Homo sapiens |
| 266 | TGFBR2 | Homo sapiens |
| 267 | TINCR | Homo sapiens |
| 268 | TMEM238L | Homo sapiens |
| 269 | TNFRSF10A-AS1 | Homo sapiens |
| 270 | TOP2A | Homo sapiens |
| 271 | TP53 | Homo sapiens |
| 272 | TP53COR1 | Homo sapiens |
| 273 | TRERNA1 | Homo sapiens |
| 274 | TSNAX-DISC1 | Homo sapiens |
| 275 | TUG1 | Homo sapiens |
| 276 | TUSC7 | Homo sapiens |
| 277 | TYMP | Homo sapiens |
| 278 | TYMS | Homo sapiens |
| 279 | UCA1 | Homo sapiens |
| 280 | UMPS | Homo sapiens |
| 281 | URGCP | Homo sapiens |
| 282 | VEGFA | Homo sapiens |
| 283 | VEGFC | Homo sapiens |
| 284 | VEGFD | Homo sapiens |
| 285 | VPS9D1-AS1 | Homo sapiens |
| 286 | VTRNA2-1 | Homo sapiens |
| 287 | WNT2B | Homo sapiens |
| 288 | WNT6 | Homo sapiens |
| 289 | WNT9A | Homo sapiens |
| 290 | WT1-AS | Homo sapiens |
| 291 | XIST | Homo sapiens |
| 292 | YAP1 | Homo sapiens |
| 293 | ZEB1-AS1 | Homo sapiens |
| 294 | ZFAS1 | Homo sapiens |
| 295 | ZFHX4-AS1 | Homo sapiens |
| 296 | ZMAT1 | Homo sapiens |
| 297 | STAT3 | Homo sapiens |
| 298 | CD274 | Homo sapiens |
| 299 | MTOR | Homo sapiens |
| 300 | BIRC5 | Homo sapiens |
| 301 | IL6 | Homo sapiens |
| 302 | CXCL8 | Homo sapiens |
| 303 | ALB | Homo sapiens |
| 304 | IL10 | Homo sapiens |
| 305 | TNF | Homo sapiens |
| 306 | IL2 | Homo sapiens |
| 307 | CXCR4 | Homo sapiens |
| 308 | JAG1 | Homo sapiens |
| 309 | NFKB1 | Homo sapiens |
| 310 | ERCC1 | Homo sapiens |
| 311 | KDR | Homo sapiens |
| 312 | CRP | Homo sapiens |
| 313 | IL17A | Homo sapiens |
| 314 | SNAI1 | Homo sapiens |
| 315 | PCNA | Homo sapiens |
| 316 | XRCC1 | Homo sapiens |
| 317 | EZH2 | Homo sapiens |
| 318 | MAPK8 | Homo sapiens |
| 319 | PROM1 | Homo sapiens |
| 320 | TNFSF10 | Homo sapiens |
| 321 | FOXP3 | Homo sapiens |
| 322 | PDCD1 | Homo sapiens |
| 323 | IFNG | Homo sapiens |
| 324 | FAS | Homo sapiens |
| 325 | TWIST1 | Homo sapiens |
| 326 | FOXM1 | Homo sapiens |
| 327 | VIM | Homo sapiens |
| 328 | NOTCH1 | Homo sapiens |
| 329 | CXCL12 | Homo sapiens |
| 330 | SPP1 | Homo sapiens |
| 331 | ERBB3 | Homo sapiens |
| 332 | MDM2 | Homo sapiens |
| 333 | RHOA | Homo sapiens |
| 334 | MAP2K7 | Homo sapiens |
| 335 | SRC | Homo sapiens |
| 336 | TLR4 | Homo sapiens |
| 337 | SOX2 | Homo sapiens |
| 338 | PIK3CB | Homo sapiens |
| 339 | SHH | Homo sapiens |
| 340 | PGC | Homo sapiens |
| 341 | ICAM1 | Homo sapiens |
| 342 | BRCA1 | Homo sapiens |
| 343 | JAK2 | Homo sapiens |
| 344 | CD8A | Homo sapiens |
| 345 | E2F1 | Homo sapiens |
| 346 | FASLG | Homo sapiens |
| 347 | SIRT1 | Homo sapiens |
| 348 | PPARG | Homo sapiens |
| 349 | MIR146A | Homo sapiens |
| 350 | GSTT1 | Homo sapiens |
| 351 | EPCAM | Homo sapiens |
| 352 | MMP1 | Homo sapiens |
| 353 | CASP9 | Homo sapiens |
| 354 | CD34 | Homo sapiens |
| 355 | CD4 | Homo sapiens |
| 356 | HPSE | Homo sapiens |
| 357 | SP1 | Homo sapiens |
| 358 | POU5F1 | Homo sapiens |
| 359 | ABCG2 | Homo sapiens |
| 360 | ATM | Homo sapiens |
| 361 | GHRL | Homo sapiens |
| 362 | LEP | Homo sapiens |
| 363 | RELA | Homo sapiens |
| 364 | PIK3CG | Homo sapiens |
| 365 | ABCC1 | Homo sapiens |
| 366 | HMGB1 | Homo sapiens |
| 367 | GLI1 | Homo sapiens |
| 368 | NODAL | Homo sapiens |
| 369 | ZEB1 | Homo sapiens |
| 370 | TIMP1 | Homo sapiens |
| 371 | NOS2 | Homo sapiens |
| 372 | CAV1 | Homo sapiens |
| 373 | MMP14 | Homo sapiens |
| 374 | LGR5 | Homo sapiens |
| 375 | MUC16 | Homo sapiens |
| 376 | MACC1 | Homo sapiens |
| 377 | SERPINE1 | Homo sapiens |
| 378 | PARP1 | Homo sapiens |
| 379 | PTK2 | Homo sapiens |
| 380 | PLAUR | Homo sapiens |
| 381 | CASP8 | Homo sapiens |
| 382 | IL4 | Homo sapiens |
| 383 | IGF1 | Homo sapiens |
| 384 | ERCC2 | Homo sapiens |
| 385 | S100A4 | Homo sapiens |
| 386 | BMI1 | Homo sapiens |
| 387 | SST | Homo sapiens |
| 388 | ANXA5 | Homo sapiens |
| 389 | MAPK14 | Homo sapiens |
| 390 | CDK4 | Homo sapiens |
| 391 | HSP90AA1 | Homo sapiens |
| 392 | PTPN11 | Homo sapiens |
| 393 | CSF3 | Homo sapiens |
| 394 | HSPA5 | Homo sapiens |
| 395 | BRAF | Homo sapiens |
| 396 | RAC1 | Homo sapiens |
| 397 | PTP4A3 | Homo sapiens |
| 398 | RASSF1 | Homo sapiens |
| 399 | HSPA4 | Homo sapiens |
| 400 | SPARC | Homo sapiens |
| 401 | IGF1R | Homo sapiens |
| 402 | OGG1 | Homo sapiens |
| 403 | EGR3 | Homo sapiens |
| 404 | ARID1A | Homo sapiens |
| 405 | MCL1 | Homo sapiens |
| 406 | MIR148A | Homo sapiens |
| 407 | MIR126 | Homo sapiens |
| 408 | ABO | Homo sapiens |
| 409 | SOX9 | Homo sapiens |
| 410 | CD68 | Homo sapiens |
| 411 | CDH2 | Homo sapiens |
| 412 | PSMD9 | Homo sapiens |
| 413 | ZEB2 | Homo sapiens |
| 414 | PRKAA1 | Homo sapiens |
| 415 | CLDN18 | Homo sapiens |
| 416 | PRNP | Homo sapiens |
| 417 | AGT | Homo sapiens |
| 418 | CYP2E1 | Homo sapiens |
| 419 | CDK6 | Homo sapiens |
| 420 | SOD2 | Homo sapiens |
| 421 | BECN1 | Homo sapiens |
| 422 | HP | Homo sapiens |
| 423 | MTDH | Homo sapiens |
| 424 | LGALS3 | Homo sapiens |
| 425 | PLK1 | Homo sapiens |
| 426 | PML | Homo sapiens |
| 427 | TIMP2 | Homo sapiens |
| 428 | FBXW7 | Homo sapiens |
| 429 | MIF | Homo sapiens |
| 430 | ADIPOQ | Homo sapiens |
| 431 | WNT5A | Homo sapiens |
| 432 | SFRP1 | Homo sapiens |
| 433 | IL11 | Homo sapiens |
| 434 | EPHA2 | Homo sapiens |
| 435 | IGF2 | Homo sapiens |
| 436 | PKM | Homo sapiens |
| 437 | IL18 | Homo sapiens |
| 438 | IL1A | Homo sapiens |
| 439 | SMAD3 | Homo sapiens |
| 440 | FLT1 | Homo sapiens |
| 441 | COL18A1 | Homo sapiens |
| 442 | GRP | Homo sapiens |
| 443 | HDAC1 | Homo sapiens |
| 444 | CAT | Homo sapiens |
| 445 | PLCE1 | Homo sapiens |
| 446 | PPP1R1B | Homo sapiens |
| 447 | TLR2 | Homo sapiens |
| 448 | KRT19 | Homo sapiens |
| 449 | HMGA2 | Homo sapiens |
| 450 | FGF2 | Homo sapiens |
| 451 | CYP1A1 | Homo sapiens |
| 452 | HNF4A | Homo sapiens |
| 453 | SLC2A1 | Homo sapiens |
| 454 | CCL2 | Homo sapiens |
| 455 | SERPINA1 | Homo sapiens |
| 456 | IL17F | Homo sapiens |
| 457 | MIR18A | Homo sapiens |
| 458 | CD40 | Homo sapiens |
| 459 | LPA | Homo sapiens |
| 460 | PDCD4 | Homo sapiens |
| 461 | THBS1 | Homo sapiens |
| 462 | EGR1 | Homo sapiens |
| 463 | MIR375 | Homo sapiens |
| 464 | IL6ST | Homo sapiens |
| 465 | SOD1 | Homo sapiens |
| 466 | OLFM4 | Homo sapiens |
| 467 | ALDH2 | Homo sapiens |
| 468 | CTSD | Homo sapiens |
| 469 | FGFR1 | Homo sapiens |
| 470 | PEBP1 | Homo sapiens |
| 471 | CSF2 | Homo sapiens |
| 472 | FOXO1 | Homo sapiens |
| 473 | CD24 | Homo sapiens |
| 474 | KDM1A | Homo sapiens |
| 475 | BRCA2 | Homo sapiens |
| 476 | CTLA4 | Homo sapiens |
| 477 | TNFRSF10B | Homo sapiens |
| 478 | MIR155 | Homo sapiens |
| 479 | XIAP | Homo sapiens |
| 480 | CREB1 | Homo sapiens |
| 481 | KLRK1 | Homo sapiens |
| 482 | SERPINB3 | Homo sapiens |
| 483 | CCNB1 | Homo sapiens |
| 484 | MIR10B | Homo sapiens |
| 485 | PTK2B | Homo sapiens |
| 486 | CD82 | Homo sapiens |
| 487 | IGFBP3 | Homo sapiens |
| 488 | FOXO3 | Homo sapiens |
| 489 | APEX1 | Homo sapiens |
| 490 | NANOG | Homo sapiens |
| 491 | CCL5 | Homo sapiens |
| 492 | GDF15 | Homo sapiens |
| 493 | LOX | Homo sapiens |
| 494 | AREG | Homo sapiens |
| 495 | EPO | Homo sapiens |
| 496 | DAPK1 | Homo sapiens |
| 497 | ERBB4 | Homo sapiens |
| 498 | KLF4 | Homo sapiens |
| 499 | DNMT3A | Homo sapiens |
| 500 | MIR204 | Homo sapiens |
| 501 | FASN | Homo sapiens |
| 502 | TIMP3 | Homo sapiens |
| 503 | XAF1 | Homo sapiens |
| 504 | BMP2 | Homo sapiens |
| 505 | F3 | Homo sapiens |
| 506 | XRCC3 | Homo sapiens |
| 507 | VIP | Homo sapiens |
| 508 | DNMT3B | Homo sapiens |
| 509 | PAK4 | Homo sapiens |
| 510 | MIR31 | Homo sapiens |
| 511 | MIR196A2 | Homo sapiens |
| 512 | PPP1R1A | Homo sapiens |
| 513 | SPHK1 | Homo sapiens |
| 514 | CXCR2 | Homo sapiens |
| 515 | MIR29C | Homo sapiens |
| 516 | ACE | Homo sapiens |
| 517 | TP73 | Homo sapiens |
| 518 | TUBB3 | Homo sapiens |
| 519 | CTSB | Homo sapiens |
| 520 | CBLB | Homo sapiens |
| 521 | EIF4E | Homo sapiens |
| 522 | WNT1 | Homo sapiens |
| 523 | PRKG1 | Homo sapiens |
| 524 | GLI2 | Homo sapiens |
| 525 | DKK1 | Homo sapiens |
| 526 | LGALS1 | Homo sapiens |
| 527 | MYCL | Homo sapiens |
| 528 | GSTK1 | Homo sapiens |
| 529 | HLA-A | Homo sapiens |
| 530 | NAT2 | Homo sapiens |
| 531 | MAP2K1 | Homo sapiens |
| 532 | CDKN3 | Homo sapiens |
| 533 | MAD2L1 | Homo sapiens |
| 534 | SKP2 | Homo sapiens |
| 535 | NAMPT | Homo sapiens |
| 536 | F2R | Homo sapiens |
| 537 | KIT | Homo sapiens |
| 538 | MIR17 | Homo sapiens |
| 539 | FOS | Homo sapiens |
| 540 | REG1A | Homo sapiens |
| 541 | ETS1 | Homo sapiens |
| 542 | MIR152 | Homo sapiens |
| 543 | CIAPIN1 | Homo sapiens |
| 544 | BSG | Homo sapiens |
| 545 | ERCC5 | Homo sapiens |
| 546 | CXCL1 | Homo sapiens |
| 547 | MSMP | Homo sapiens |
| 548 | HAVCR2 | Homo sapiens |
| 549 | NRP1 | Homo sapiens |
| 550 | CDC42 | Homo sapiens |
| 551 | KRT7 | Homo sapiens |
| 552 | MIR7-3HG | Homo sapiens |
| 553 | KIAA1524 | Homo sapiens |
| 554 | CDK1 | Homo sapiens |
| 555 | PTCH1 | Homo sapiens |
| 556 | STK11 | Homo sapiens |
| 557 | MIR149 | Homo sapiens |
| 558 | AQP3 | Homo sapiens |
| 559 | S100A6 | Homo sapiens |
| 560 | SERPINE2 | Homo sapiens |
| 561 | ARHGDIB | Homo sapiens |
| 562 | TBX21 | Homo sapiens |
| 563 | MIR22 | Homo sapiens |
| 564 | VTCN1 | Homo sapiens |
| 565 | ST3GAL4 | Homo sapiens |
| 566 | PDCD1LG2 | Homo sapiens |
| 567 | CHEK1 | Homo sapiens |
| 568 | MPO | Homo sapiens |
| 569 | MAP1LC3A | Homo sapiens |
| 570 | ANGPT2 | Homo sapiens |
| 571 | ZNRD1 | Homo sapiens |
| 572 | IL2RA | Homo sapiens |
| 573 | CHEK2 | Homo sapiens |
| 574 | GDE1 | Homo sapiens |
| 575 | AGER | Homo sapiens |
| 576 | FUT4 | Homo sapiens |
| 577 | MIR195 | Homo sapiens |
| 578 | CD14 | Homo sapiens |
| 579 | MIR335 | Homo sapiens |
| 580 | LTA | Homo sapiens |
| 581 | KLF5 | Homo sapiens |
| 582 | POSTN | Homo sapiens |
| 583 | FGFR4 | Homo sapiens |
| 584 | SALL4 | Homo sapiens |
| 585 | YBX1 | Homo sapiens |
| 586 | TNFSF13 | Homo sapiens |
| 587 | MIR183 | Homo sapiens |
| 588 | MDK | Homo sapiens |
| 589 | SATB1 | Homo sapiens |
| 590 | MT2A | Homo sapiens |
| 591 | SCGB2A1 | Homo sapiens |
| 592 | TXN | Homo sapiens |
| 593 | CCR7 | Homo sapiens |
| 594 | STMN1 | Homo sapiens |
| 595 | KISS1 | Homo sapiens |
| 596 | ANXA2 | Homo sapiens |
| 597 | LILRB1 | Homo sapiens |
| 598 | TOP1 | Homo sapiens |
| 599 | CYR61 | Homo sapiens |
| 600 | ITGB1 | Homo sapiens |
| 601 | AHR | Homo sapiens |
| 602 | SOX4 | Homo sapiens |
| 603 | DICER1 | Homo sapiens |
| 604 | HSPD1 | Homo sapiens |
| 605 | RPRM | Homo sapiens |
| 606 | PRKCA | Homo sapiens |
| 607 | CEACAM6 | Homo sapiens |
| 608 | KRT18 | Homo sapiens |
| 609 | CCNE1 | Homo sapiens |
| 610 | MKI67 | Homo sapiens |
| 611 | CD79A | Homo sapiens |
| 612 | ZKSCAN1 | Homo sapiens |
| 613 | MIR133B | Homo sapiens |
| 614 | RETN | Homo sapiens |
| 615 | CCK | Homo sapiens |
| 616 | MIR206 | Homo sapiens |
| 617 | CTGF | Homo sapiens |
| 618 | MMP11 | Homo sapiens |
| 619 | TNFRSF10A | Homo sapiens |
| 620 | DDIT3 | Homo sapiens |
| 621 | BBC3 | Homo sapiens |
| 622 | NEDD9 | Homo sapiens |
| 623 | LYVE1 | Homo sapiens |
| 624 | ADAM9 | Homo sapiens |
| 625 | GATA6 | Homo sapiens |
| 626 | STAT1 | Homo sapiens |
| 627 | ODC1 | Homo sapiens |
| 628 | EZR | Homo sapiens |
| 629 | HPGD | Homo sapiens |
| 630 | CHFR | Homo sapiens |
| 631 | GJA1 | Homo sapiens |
| 632 | ADAM17 | Homo sapiens |
| 633 | MMP13 | Homo sapiens |
| 634 | ENO1 | Homo sapiens |
| 635 | SOCS1 | Homo sapiens |
| 636 | PTPA | Homo sapiens |
| 637 | NDRG1 | Homo sapiens |
| 638 | GPX3 | Homo sapiens |
| 639 | WNT2 | Homo sapiens |
| 640 | MIR125A | Homo sapiens |
| 641 | CD40LG | Homo sapiens |
| 642 | FAT4 | Homo sapiens |
| 643 | MIR23A | Homo sapiens |
| 644 | MECP2 | Homo sapiens |
| 645 | NOD1 | Homo sapiens |
| 646 | RUNX1 | Homo sapiens |
| 647 | ALDH1A1 | Homo sapiens |
| 648 | MSH6 | Homo sapiens |
| 649 | CD163 | Homo sapiens |
| 650 | NCL | Homo sapiens |
| 651 | TLR9 | Homo sapiens |
| 652 | AR | Homo sapiens |
| 653 | CFL1 | Homo sapiens |
| 654 | FZD7 | Homo sapiens |
| 655 | DCC | Homo sapiens |
| 656 | PDGFRB | Homo sapiens |
| 657 | RACK1 | Homo sapiens |
| 658 | MUC4 | Homo sapiens |
| 659 | MIR17HG | Homo sapiens |
| 660 | TERC | Homo sapiens |
| 661 | MIR19A | Homo sapiens |
| 662 | CTSL | Homo sapiens |
| 663 | SDC1 | Homo sapiens |
| 664 | ROS1 | Homo sapiens |
| 665 | XRCC6 | Homo sapiens |
| 666 | TNFRSF6B | Homo sapiens |
| 667 | RB1 | Homo sapiens |
| 668 | LDHA | Homo sapiens |
| 669 | ERCC4 | Homo sapiens |
| 670 | MIR200B | Homo sapiens |
| 671 | HDAC2 | Homo sapiens |
| 672 | AICDA | Homo sapiens |
| 673 | MUC3A | Homo sapiens |
| 674 | FAP | Homo sapiens |
| 675 | PTGER4 | Homo sapiens |
| 676 | GATA3 | Homo sapiens |
| 677 | NTN1 | Homo sapiens |
| 678 | VAV3 | Homo sapiens |
| 679 | RARA | Homo sapiens |
| 680 | MMP3 | Homo sapiens |
| 681 | AKT2 | Homo sapiens |
| 682 | CCND2 | Homo sapiens |
| 683 | BCL2L11 | Homo sapiens |
| 684 | IFNA1 | Homo sapiens |
| 685 | XCL1 | Homo sapiens |
| 686 | NFE2L2 | Homo sapiens |
| 687 | HBEGF | Homo sapiens |
| 688 | LCN2 | Homo sapiens |
| 689 | ACKR3 | Homo sapiens |
| 690 | ATG5 | Homo sapiens |
| 691 | NOTCH2 | Homo sapiens |
| 692 | PGF | Homo sapiens |
| 693 | DKK3 | Homo sapiens |
| 694 | SAPCD2 | Homo sapiens |
| 695 | CD80 | Homo sapiens |
| 696 | RPSA | Homo sapiens |
| 697 | ROCK1 | Homo sapiens |
| 698 | CRKL | Homo sapiens |
| 699 | BUB1B | Homo sapiens |
| 700 | HMOX1 | Homo sapiens |
| 701 | IL32 | Homo sapiens |
| 702 | SQSTM1 | Homo sapiens |
| 703 | CASP7 | Homo sapiens |
| 704 | CLIC1 | Homo sapiens |
| 705 | MIR27B | Homo sapiens |
| 706 | THY1 | Homo sapiens |
| 707 | GATA4 | Homo sapiens |
| 708 | SIRT3 | Homo sapiens |
| 709 | MAGEA3 | Homo sapiens |
| 710 | MTA1 | Homo sapiens |
| 711 | ALCAM | Homo sapiens |
| 712 | POLB | Homo sapiens |
| 713 | WNT3A | Homo sapiens |
| 714 | MGAT5 | Homo sapiens |
| 715 | COL1A1 | Homo sapiens |
| 716 | CDKN1C | Homo sapiens |
| 717 | CYP19A1 | Homo sapiens |
| 718 | DDX53 | Homo sapiens |
| 719 | ERAS | Homo sapiens |
| 720 | PXN | Homo sapiens |
| 721 | CTTN | Homo sapiens |
| 722 | GSK3B | Homo sapiens |
| 723 | NDRG2 | Homo sapiens |
| 724 | BIRC7 | Homo sapiens |
| 725 | SRI | Homo sapiens |
| 726 | CCR4 | Homo sapiens |
| 727 | VDR | Homo sapiens |
| 728 | ANXA7 | Homo sapiens |
| 729 | COL1A2 | Homo sapiens |
| 730 | DCTN6 | Homo sapiens |
| 731 | MIR130A | Homo sapiens |
| 732 | BDNF | Homo sapiens |
| 733 | CXCR3 | Homo sapiens |
| 734 | CYP2A6 | Homo sapiens |
| 735 | HSP90B1 | Homo sapiens |
| 736 | GRB7 | Homo sapiens |
| 737 | MME | Homo sapiens |
| 738 | SNAI2 | Homo sapiens |
| 739 | PROX1 | Homo sapiens |
| 740 | MYCN | Homo sapiens |
| 741 | RPS6KB1 | Homo sapiens |
| 742 | MSI1 | Homo sapiens |
| 743 | TXNRD1 | Homo sapiens |
| 744 | CCR2 | Homo sapiens |
| 745 | EIF5A2 | Homo sapiens |
| 746 | MIR144 | Homo sapiens |
| 747 | TIAM1 | Homo sapiens |
| 748 | ESM1 | Homo sapiens |
| 749 | MIRLET7B | Homo sapiens |
| 750 | INHBA | Homo sapiens |
| 751 | FEN1 | Homo sapiens |
| 752 | CLDN1 | Homo sapiens |
| 753 | S100A9 | Homo sapiens |
| 754 | IL23A | Homo sapiens |
| 755 | NANOGP8 | Homo sapiens |
| 756 | NOD2 | Homo sapiens |
| 757 | ENG | Homo sapiens |
| 758 | NCAM1 | Homo sapiens |
| 759 | NR4A1 | Homo sapiens |
| 760 | E2F3 | Homo sapiens |
| 761 | ANGPT1 | Homo sapiens |
| 762 | SMAD7 | Homo sapiens |
| 763 | NRAS | Homo sapiens |
| 764 | STARD3 | Homo sapiens |
| 765 | ABCC3 | Homo sapiens |
| 766 | TOB1 | Homo sapiens |
| 767 | BMP4 | Homo sapiens |
| 768 | ARL6IP5 | Homo sapiens |
| 769 | PCLAF | Homo sapiens |
| 770 | FSCN1 | Homo sapiens |
| 771 | IDO1 | Homo sapiens |
| 772 | MIR185 | Homo sapiens |
| 773 | MSLN | Homo sapiens |
| 774 | CFLAR | Homo sapiens |
| 775 | BAK1 | Homo sapiens |
| 776 | CDK5 | Homo sapiens |
| 777 | PGR | Homo sapiens |
| 778 | TERF2 | Homo sapiens |
| 779 | MTA2 | Homo sapiens |
| 780 | NTRK2 | Homo sapiens |
| 781 | ADIPOR1 | Homo sapiens |
| 782 | TEK | Homo sapiens |
| 783 | TET1 | Homo sapiens |
| 784 | MIR34B | Homo sapiens |
| 785 | RNMT | Homo sapiens |
| 786 | MYH9 | Homo sapiens |
| 787 | XRCC5 | Homo sapiens |
| 788 | MIR34C | Homo sapiens |
| 789 | MIR182 | Homo sapiens |
| 790 | WT1 | Homo sapiens |
| 791 | MIR137 | Homo sapiens |
| 792 | LIN28A | Homo sapiens |
| 793 | KDM4B | Homo sapiens |
| 794 | HOXA10 | Homo sapiens |
| 795 | PFKFB3 | Homo sapiens |
| 796 | PTHLH | Homo sapiens |
| 797 | ALPP | Homo sapiens |
| 798 | PLA2G2A | Homo sapiens |
| 799 | GIF | Homo sapiens |
| 800 | UCHL1 | Homo sapiens |
| 801 | MIR429 | Homo sapiens |
| 802 | BUB1 | Homo sapiens |
| 803 | MICA | Homo sapiens |
| 804 | MIR217 | Homo sapiens |
| 805 | KLK6 | Homo sapiens |
| 806 | SPINK1 | Homo sapiens |
| 807 | HOXC10 | Homo sapiens |
| 808 | NFKBIA | Homo sapiens |
| 809 | CD9 | Homo sapiens |
| 810 | YWHAZ | Homo sapiens |
| 811 | CBL | Homo sapiens |
| 812 | PTPN6 | Homo sapiens |
| 813 | ZFHX3 | Homo sapiens |
| 814 | ADH1B | Homo sapiens |
| 815 | TCHP | Homo sapiens |
| 816 | KCNH2 | Homo sapiens |
| 817 | TFAP4 | Homo sapiens |
| 818 | NOS3 | Homo sapiens |
| 819 | HDGF | Homo sapiens |
| 820 | AURKB | Homo sapiens |
| 821 | MAP1LC3B | Homo sapiens |
| 822 | PCDH10 | Homo sapiens |
| 823 | SERPINB2 | Homo sapiens |
| 824 | BCL2L2 | Homo sapiens |
| 825 | KLF8 | Homo sapiens |
| 826 | HK1 | Homo sapiens |
| 827 | HOXD10 | Homo sapiens |
| 828 | LEPR | Homo sapiens |
| 829 | BRD4 | Homo sapiens |
| 830 | SIAH1 | Homo sapiens |
| 831 | HMGB3 | Homo sapiens |
| 832 | CRK | Homo sapiens |
| 833 | PGK1 | Homo sapiens |
| 834 | RNF180 | Homo sapiens |
| 835 | F2RL1 | Homo sapiens |
| 836 | LYPD5 | Homo sapiens |
| 837 | SMOX | Homo sapiens |
| 838 | PECAM1 | Homo sapiens |
| 839 | PNP | Homo sapiens |
| 840 | PRKCB | Homo sapiens |
| 841 | CCR5 | Homo sapiens |
| 842 | IL22 | Homo sapiens |
| 843 | MIR29A | Homo sapiens |
| 844 | HTRA1 | Homo sapiens |
| 845 | SOCS3 | Homo sapiens |
| 846 | KDM5A | Homo sapiens |
| 847 | TES | Homo sapiens |
| 848 | FN1 | Homo sapiens |
| 849 | WWTR1 | Homo sapiens |
| 850 | KLK10 | Homo sapiens |
| 851 | EIF4EBP1 | Homo sapiens |
| 852 | MIR449A | Homo sapiens |
| 853 | VCAM1 | Homo sapiens |
| 854 | CTNND1 | Homo sapiens |
| 855 | CMA1 | Homo sapiens |
| 856 | S100P | Homo sapiens |
| 857 | RECK | Homo sapiens |
| 858 | RASAL1 | Homo sapiens |
| 859 | ID1 | Homo sapiens |
| 860 | TP63 | Homo sapiens |
| 861 | UHRF1 | Homo sapiens |
| 862 | MORC2 | Homo sapiens |
| 863 | TSPAN8 | Homo sapiens |
| 864 | PHB2 | Homo sapiens |
| 865 | L1CAM | Homo sapiens |
| 866 | FST | Homo sapiens |
| 867 | BMP7 | Homo sapiens |
| 868 | SLC4A1 | Homo sapiens |
| 869 | HDAC9 | Homo sapiens |
| 870 | TMEFF2 | Homo sapiens |
| 871 | MS4A1 | Homo sapiens |
| 872 | SULF1 | Homo sapiens |
| 873 | SMO | Homo sapiens |
| 874 | PDPN | Homo sapiens |
| 875 | VGLL4 | Homo sapiens |
| 876 | PHB | Homo sapiens |
| 877 | EIF3A | Homo sapiens |
| 878 | TLR7 | Homo sapiens |
| 879 | RHOC | Homo sapiens |
| 880 | CLU | Homo sapiens |
| 881 | XPC | Homo sapiens |
| 882 | ESR1 | Homo sapiens |
| 883 | ANGPTL4 | Homo sapiens |
| 884 | MIR494 | Homo sapiens |
| 885 | METTL3 | Homo sapiens |
| 886 | BCL6B | Homo sapiens |
| 887 | TMPRSS4 | Homo sapiens |
| 888 | HK2 | Homo sapiens |
| 889 | ANO1 | Homo sapiens |
| 890 | CMTM3 | Homo sapiens |
| 891 | B3GAT1 | Homo sapiens |
| 892 | MYD88 | Homo sapiens |
| 893 | PTH | Homo sapiens |
| 894 | FOSL1 | Homo sapiens |
| 895 | ILK | Homo sapiens |
| 896 | EP300 | Homo sapiens |
| 897 | PAQR3 | Homo sapiens |
| 898 | SLC1A5 | Homo sapiens |
| 899 | MIR30A | Homo sapiens |
| 900 | FOXA1 | Homo sapiens |
| 901 | REG3A | Homo sapiens |
| 902 | PTPN1 | Homo sapiens |
| 903 | STC2 | Homo sapiens |
| 904 | CD276 | Homo sapiens |
| 905 | AKR1B10 | Homo sapiens |
| 906 | BTG2 | Homo sapiens |
| 907 | TLR5 | Homo sapiens |
| 908 | TF | Homo sapiens |
| 909 | MIR503 | Homo sapiens |
| 910 | CASC11 | Homo sapiens |
| 911 | XPO1 | Homo sapiens |
| 912 | IFNA2 | Homo sapiens |
| 913 | DROSHA | Homo sapiens |
| 914 | KITLG | Homo sapiens |
| 915 | EPOR | Homo sapiens |
| 916 | PTGES | Homo sapiens |
| 917 | TACSTD2 | Homo sapiens |
| 918 | CDH11 | Homo sapiens |
| 919 | CADM1 | Homo sapiens |
| 920 | LGMN | Homo sapiens |
| 921 | ADIPOR2 | Homo sapiens |
| 922 | ATF4 | Homo sapiens |
| 923 | COX8A | Homo sapiens |
| 924 | SRF | Homo sapiens |
| 925 | CD83 | Homo sapiens |
| 926 | MAP3K5 | Homo sapiens |
| 927 | IL24 | Homo sapiens |
| 928 | MIR497 | Homo sapiens |
| 929 | GJB1 | Homo sapiens |
| 930 | ANGPTL2 | Homo sapiens |
| 931 | TRIM59 | Homo sapiens |
| 932 | ADAM10 | Homo sapiens |
| 933 | SMYD3 | Homo sapiens |
| 934 | HES1 | Homo sapiens |
| 935 | RASSF10 | Homo sapiens |
| 936 | HSPB1 | Homo sapiens |
| 937 | RUNX2 | Homo sapiens |
| 938 | ASPN | Homo sapiens |
| 939 | GCG | Homo sapiens |
| 940 | MMP10 | Homo sapiens |
| 941 | STK16 | Homo sapiens |
| 942 | CD151 | Homo sapiens |
| 943 | DAP | Homo sapiens |
| 944 | CXCR1 | Homo sapiens |
| 945 | GOLPH3 | Homo sapiens |
| 946 | HHIP | Homo sapiens |
| 947 | MIR139 | Homo sapiens |
| 948 | MIR99A | Homo sapiens |
| 949 | FOXQ1 | Homo sapiens |
| 950 | APOB | Homo sapiens |
| 951 | TNFAIP8L2 | Homo sapiens |
| 952 | TNFRSF25 | Homo sapiens |
| 953 | USP22 | Homo sapiens |
| 954 | CT83 | Homo sapiens |
| 955 | DMBT1 | Homo sapiens |
| 956 | CBR1 | Homo sapiens |
| 957 | NQO1 | Homo sapiens |
| 958 | HSPB3 | Homo sapiens |
| 959 | ECM1 | Homo sapiens |
| 960 | MAPK9 | Homo sapiens |
| 961 | HMGA1 | Homo sapiens |
| 962 | DIABLO | Homo sapiens |
| 963 | LIN28B | Homo sapiens |
| 964 | FADD | Homo sapiens |
| 965 | MERTK | Homo sapiens |
| 966 | GNL3 | Homo sapiens |
| 967 | LGALS9 | Homo sapiens |
| 968 | HER2 | Homo sapiens |
| 969 | PDGFRA | Homo sapiens |
| 970 | Candi TMP1 | Homo sapiens |
| 971 | HSP90A | Homo sapiens |
| 972 | RRM2 | Homo sapiens |
| 973 | ADRB2 | Homo sapiens |
| 974 | IMPDH1 | Homo sapiens |
| 975 | PD-L1 | Homo sapiens |
| 976 | PD-1 | Homo sapiens |
| 977 | TOP2 | Homo sapiens |
| 978 | GPR119 | Homo sapiens |
| 979 | hDNA | Homo sapiens |
| 980 | MMP-9 | Homo sapiens |
| 981 | CD66e | Homo sapiens |
| 982 | CD55 | Homo sapiens |
| 983 | HSP70 | Homo sapiens |
| 984 | LAG3 | Homo sapiens |
| 985 | NaC | Homo sapiens |
| 986 | PDGFR | Homo sapiens |
| 987 | PTK | Homo sapiens |
| 988 | VEGFR | Homo sapiens |
| 989 | HYAL | Homo sapiens |
| 990 | PARP | Homo sapiens |

**Table S3**

The GO terms of therapy target genes and their corresponding count, Corrected *p*-values, and gene count.

| **GO Term Name** | **GO Term ID** | **Corrected**  ***P*-value** | **Genes Count** |
| --- | --- | --- | --- |
| protein autophosphorylation | GO:0046777 | 4.32E-12 | 8 |
| catabolic process | GO:0009056 | 7.53E-12 | 13 |
| protein phosphorylation | GO:0006468 | 3.31E-11 | 12 |
| negative regulation of programmed cell death | GO:0043069 | 1.13E-10 | 10 |
| negative regulation of metabolic process | GO:0009892 | 2.13E-10 | 13 |
| negative regulation of cell death | GO:0060548 | 2.70E-10 | 10 |
| phosphorylation | GO:0016310 | 3.62E-10 | 12 |
| response to oxygen-containing compound | GO:1901700 | 3.69E-10 | 11 |
| cellular response to oxygen-containing compound | GO:1901701 | 5.45E-10 | 10 |
| regulation of cell death | GO:0010941 | 7.38E-10 | 11 |
| negative regulation of cellular process | GO:0048523 | 7.82E-10 | 14 |
| peptidyl-amino acid modification | GO:0018193 | 8.73E-10 | 10 |
| positive regulation of cellular process | GO:0048522 | 2.55E-09 | 14 |
| regulation of localization | GO:0032879 | 2.96E-09 | 12 |
| cellular response to stress | GO:0033554 | 3.66E-09 | 11 |
| negative regulation of biological process | GO:0048519 | 4.95E-09 | 14 |
| negative regulation of apoptotic process | GO:0043066 | 7.16E-09 | 9 |
| protein metabolic process | GO:0019538 | 8.72E-09 | 14 |
| regulation of response to stimulus | GO:0048583 | 9.29E-09 | 13 |
| cellular response to oxidative stress | GO:0034599 | 9.91E-09 | 7 |
| phosphatidylinositol 3-kinase signaling | GO:0014065 | 1.19E-08 | 6 |
| cell death | GO:0008219 | 1.48E-08 | 11 |
| regulation of cell motility | GO:2000145 | 1.50E-08 | 9 |
| cellular response to chemical stimulus | GO:0070887 | 1.65E-08 | 12 |
| phosphate-containing compound metabolic process | GO:0006796 | 1.74E-08 | 12 |
| positive regulation of biological process | GO:0048518 | 1.75E-08 | 14 |
| phosphorus metabolic process | GO:0006793 | 1.90E-08 | 12 |
| regulation of programmed cell death | GO:0043067 | 1.99E-08 | 10 |
| response to nitrogen compound | GO:1901698 | 2.07E-08 | 9 |
| cellular response to nitrogen compound | GO:1901699 | 2.47E-08 | 8 |
| regulation of locomotion | GO:0040012 | 2.72E-08 | 9 |
| phosphatidylinositol-mediated signaling | GO:0048015 | 2.80E-08 | 6 |
| cellular response to reactive oxygen species | GO:0034614 | 3.02E-08 | 6 |
| regulation of cellular component movement | GO:0051270 | 3.06E-08 | 9 |
| inositol lipid-mediated signaling | GO:0048017 | 3.13E-08 | 6 |
| signaling | GO:0023052 | 3.22E-08 | 14 |
| cell communication | GO:0007154 | 3.39E-08 | 14 |
| positive regulation of metabolic process | GO:0009893 | 3.57E-08 | 12 |
| response to chemical | GO:0042221 | 3.82E-08 | 13 |
| negative regulation of cellular metabolic process | GO:0031324 | 4.89E-08 | 11 |
| intracellular signal transduction | GO:0035556 | 8.78E-08 | 11 |
| peptidyl-tyrosine autophosphorylation | GO:0038083 | 8.91E-08 | 4 |
| organonitrogen compound metabolic process | GO:1901564 | 9.18E-08 | 14 |
| cellular protein metabolic process | GO:0044267 | 9.47E-08 | 13 |
| response to stimulus | GO:0050896 | 1.12E-07 | 15 |
| response to oxidative stress | GO:0006979 | 1.69E-07 | 7 |
| response to stress | GO:0006950 | 2.08E-07 | 12 |
| cellular protein modification process | GO:0006464 | 2.10E-07 | 12 |
| protein modification process | GO:0036211 | 2.10E-07 | 12 |
| cellular response to stimulus | GO:0051716 | 2.25E-07 | 14 |
| response to reactive oxygen species | GO:0000302 | 2.32E-07 | 6 |
| cell surface receptor signaling pathway | GO:0007166 | 2.55E-07 | 11 |
| peptidyl-serine phosphorylation | GO:0018105 | 2.58E-07 | 6 |
| autophagy | GO:0006914 | 2.59E-07 | 7 |
| process utilizing autophagic mechanism | GO:0061919 | 2.59E-07 | 7 |
| programmed cell death | GO:0012501 | 3.44E-07 | 10 |
| response to peptide | GO:1901652 | 3.56E-07 | 7 |
| macromolecule modification | GO:0043412 | 3.56E-07 | 12 |
| peptidyl-serine modification | GO:0018209 | 4.35E-07 | 6 |
| regulation of cell migration | GO:0030334 | 5.09E-07 | 8 |
| positive regulation of macromolecule metabolic process | GO:0010604 | 5.19E-07 | 11 |
| signal transduction | GO:0007165 | 5.25E-07 | 13 |
| positive regulation of cell migration | GO:0030335 | 5.37E-07 | 7 |
| response to inorganic substance | GO:0010035 | 5.37E-07 | 7 |
| primary metabolic process | GO:0044238 | 5.70E-07 | 15 |
| regulation of catabolic process | GO:0009894 | 5.95E-07 | 8 |
| response to organonitrogen compound | GO:0010243 | 6.31E-07 | 8 |
| cellular metabolic process | GO:0044237 | 6.36E-07 | 15 |
| protein kinase B signaling | GO:0043491 | 6.44E-07 | 6 |
| positive regulation of cell motility | GO:2000147 | 7.42E-07 | 7 |
| regulation of cellular metabolic process | GO:0031323 | 7.69E-07 | 13 |
| regulation of apoptotic process | GO:0042981 | 7.81E-07 | 9 |
| positive regulation of cellular component movement | GO:0051272 | 9.06E-07 | 7 |
| regulation of cell-matrix adhesion | GO:0001952 | 1.00E-06 | 5 |
| positive regulation of locomotion | GO:0040017 | 1.04E-06 | 7 |
| organic substance metabolic process | GO:0071704 | 1.20E-06 | 15 |
| regulation of cellular component organization | GO:0051128 | 1.23E-06 | 10 |
| regulation of response to stress | GO:0080134 | 1.31E-06 | 9 |
| cell motility | GO:0048870 | 1.44E-06 | 9 |
| localization of cell | GO:0051674 | 1.44E-06 | 9 |
| regulation of cell communication | GO:0010646 | 1.47E-06 | 11 |
| regulation of autophagy | GO:0010506 | 1.57E-06 | 6 |
| proteolysis | GO:0006508 | 1.63E-06 | 9 |
| regulation of signaling | GO:0023051 | 1.70E-06 | 11 |
| localization | GO:0051179 | 2.01E-06 | 13 |
| metabolic process | GO:0008152 | 2.41E-06 | 15 |
| positive regulation of proteolysis | GO:0045862 | 2.49E-06 | 6 |
| positive regulation of cellular catabolic process | GO:0031331 | 2.86E-06 | 6 |
| transmembrane receptor protein tyrosine kinase signaling pathway | GO:0007169 | 2.98E-06 | 7 |
| regulation of metabolic process | GO:0019222 | 3.16E-06 | 13 |
| peptidyl-tyrosine phosphorylation | GO:0018108 | 3.34E-06 | 6 |
| peptidyl-tyrosine modification | GO:0018212 | 3.51E-06 | 6 |
| regulation of intracellular signal transduction | GO:1902531 | 3.59E-06 | 9 |
| cellular response to drug | GO:0035690 | 3.69E-06 | 6 |
| regulation of biological quality | GO:0065008 | 3.82E-06 | 11 |
| locomotion | GO:0040011 | 4.48E-06 | 9 |
| regulation of cellular response to stress | GO:0080135 | 4.72E-06 | 7 |
| apoptotic process | GO:0006915 | 6.21E-06 | 9 |
| macromolecule metabolic process | GO:0043170 | 6.37E-06 | 14 |
| negative regulation of macromolecule metabolic process | GO:0010605 | 6.66E-06 | 10 |
| positive regulation of catabolic process | GO:0009896 | 6.72E-06 | 6 |
| response to amyloid-beta | GO:1904645 | 7.79E-06 | 4 |
| positive regulation of nitrogen compound metabolic process | GO:0051173 | 8.77E-06 | 10 |
| movement of cell or subcellular component | GO:0006928 | 1.01E-05 | 9 |
| regulation of cellular catabolic process | GO:0031329 | 1.25E-05 | 7 |
| cellular catabolic process | GO:0044248 | 1.26E-05 | 9 |
| regulation of signal transduction | GO:0009966 | 1.35E-05 | 10 |
| regulation of nitrogen compound metabolic process | GO:0051171 | 1.36E-05 | 12 |
| positive regulation of cellular metabolic process | GO:0031325 | 1.38E-05 | 10 |
| regulation of cell-substrate adhesion | GO:0010810 | 1.51E-05 | 5 |
| nitrogen compound metabolic process | GO:0006807 | 1.55E-05 | 14 |
| regulation of primary metabolic process | GO:0080090 | 1.92E-05 | 12 |
| cell-matrix adhesion | GO:0007160 | 1.96E-05 | 5 |
| MAPK cascade | GO:0000165 | 1.97E-05 | 7 |
| signal transduction by protein phosphorylation | GO:0023014 | 2.03E-05 | 7 |
| cellular component organization | GO:0016043 | 2.07E-05 | 12 |
| positive regulation of cellular protein metabolic process | GO:0032270 | 2.15E-05 | 8 |
| response to organic substance | GO:0010033 | 2.17E-05 | 10 |
| regulation of cellular component biogenesis | GO:0044087 | 2.28E-05 | 7 |
| regulation of protein binding | GO:0043393 | 2.31E-05 | 5 |
| regulation of transferase activity | GO:0051338 | 2.32E-05 | 7 |
| cell migration | GO:0016477 | 2.34E-05 | 8 |
| cellular component organization or biogenesis | GO:0071840 | 2.75E-05 | 12 |
| negative regulation of nitrogen compound metabolic process | GO:0051172 | 2.78E-05 | 9 |
| growth | GO:0040007 | 2.90E-05 | 7 |
| cellular macromolecule metabolic process | GO:0044260 | 2.92E-05 | 13 |
| positive regulation of cell proliferation | GO:0008284 | 2.98E-05 | 7 |
| regulation of macromolecule metabolic process | GO:0060255 | 3.07E-05 | 12 |
| positive regulation of protein metabolic process | GO:0051247 | 3.42E-05 | 8 |
| response to external stimulus | GO:0009605 | 3.53E-05 | 9 |
| regulation of protein kinase B signaling | GO:0051896 | 3.72E-05 | 5 |
| regulation of cyclin-dependent protein serine/threonine kinase activity | GO:0000079 | 3.78E-05 | 4 |
| enzyme linked receptor protein signaling pathway | GO:0007167 | 3.79E-05 | 7 |
| regulation of cellular process | GO:0050794 | 3.95E-05 | 14 |
| animal organ development | GO:0048513 | 4.36E-05 | 10 |
| positive regulation of ubiquitin-dependent protein catabolic process | GO:2000060 | 4.59E-05 | 4 |
| regulation of cyclin-dependent protein kinase activity | GO:1904029 | 4.82E-05 | 4 |
| negative regulation of response to stimulus | GO:0048585 | 4.96E-05 | 8 |
| cellular response to organonitrogen compound | GO:0071417 | 5.12E-05 | 6 |
| positive regulation of molecular function | GO:0044093 | 5.30E-05 | 8 |
| regulation of cellular protein metabolic process | GO:0032268 | 5.56E-05 | 9 |
| regulation of phosphate metabolic process | GO:0019220 | 6.19E-05 | 8 |
| regulation of phosphorus metabolic process | GO:0051174 | 6.22E-05 | 8 |
| negative regulation of transferase activity | GO:0051348 | 6.66E-05 | 5 |
| positive regulation of cell communication | GO:0010647 | 7.00E-05 | 8 |
| positive regulation of signaling | GO:0023056 | 7.20E-05 | 8 |
| regulation of protein modification process | GO:0031399 | 7.64E-05 | 8 |
| cellular response to organic substance | GO:0071310 | 7.96E-05 | 9 |
| regulation of epithelial cell migration | GO:0010632 | 9.17E-05 | 5 |
| positive regulation of cellular protein localization | GO:1903829 | 9.98E-05 | 5 |
| negative regulation of protein metabolic process | GO:0051248 | 1.03E-04 | 7 |
| regulation of biological process | GO:0050789 | 1.06E-04 | 14 |
| regulation of organelle organization | GO:0033043 | 1.12E-04 | 7 |
| cellular component assembly | GO:0022607 | 1.14E-04 | 9 |
| regulation of macromolecule biosynthetic process | GO:0010556 | 1.20E-04 | 10 |
| positive regulation of proteolysis involved in cellular protein catabolic process | GO:1903052 | 1.26E-04 | 4 |
| regulation of protein metabolic process | GO:0051246 | 1.27E-04 | 9 |
| regulation of protein localization to nucleus | GO:1900180 | 1.30E-04 | 4 |
| regulation of cell adhesion | GO:0030155 | 1.40E-04 | 6 |
| cell-substrate adhesion | GO:0031589 | 1.54E-04 | 5 |
| regulation of molecular function | GO:0065009 | 1.54E-04 | 9 |
| regulation of phosphatidylinositol 3-kinase signaling | GO:0014066 | 1.67E-04 | 4 |
| negative regulation of cellular component organization | GO:0051129 | 1.69E-04 | 6 |
| cellular component biogenesis | GO:0044085 | 2.05E-04 | 9 |
| biological regulation | GO:0065007 | 2.16E-04 | 14 |
| negative regulation of gene expression | GO:0010629 | 2.19E-04 | 8 |
| epithelium development | GO:0060429 | 2.19E-04 | 7 |
| cell proliferation | GO:0008283 | 2.22E-04 | 8 |
| tissue development | GO:0009888 | 2.24E-04 | 8 |
| regulation of biosynthetic process | GO:0009889 | 2.30E-04 | 10 |
| epithelial cell migration | GO:0010631 | 2.37E-04 | 5 |
| ossification | GO:0001503 | 2.37E-04 | 5 |
| positive regulation of cyclin-dependent protein serine/threonine kinase activity | GO:0045737 | 2.42E-04 | 3 |
| regulation of protein kinase activity | GO:0045859 | 2.45E-04 | 6 |
| epithelium migration | GO:0090132 | 2.47E-04 | 5 |
| positive regulation of cellular protein catabolic process | GO:1903364 | 2.55E-04 | 4 |
| regulation of protein catabolic process | GO:0042176 | 2.58E-04 | 5 |
| cellular process | GO:0009987 | 2.65E-04 | 15 |
| tissue migration | GO:0090130 | 2.68E-04 | 5 |
| positive regulation of catalytic activity | GO:0043085 | 2.80E-04 | 7 |
| cellular response to peptide | GO:1901653 | 2.87E-04 | 5 |
| cell adhesion | GO:0007155 | 3.06E-04 | 7 |
| regulation of binding | GO:0051098 | 3.16E-04 | 5 |
| biological adhesion | GO:0022610 | 3.17E-04 | 7 |
| regulation of protein phosphorylation | GO:0001932 | 3.24E-04 | 7 |
| regulation of proteolysis | GO:0030162 | 3.33E-04 | 6 |
| regulation of ubiquitin-dependent protein catabolic process | GO:2000058 | 3.44E-04 | 4 |
| reproductive process | GO:0022414 | 3.71E-04 | 7 |
| positive regulation of cyclin-dependent protein kinase activity | GO:1904031 | 3.71E-04 | 3 |
| reproduction | GO:0000003 | 3.73E-04 | 7 |
| regulation of catalytic activity | GO:0050790 | 3.88E-04 | 8 |
| positive regulation of cell adhesion | GO:0045785 | 3.94E-04 | 5 |
| plasma membrane bounded cell projection organization | GO:0120036 | 3.96E-04 | 7 |
| multicellular organismal process | GO:0032501 | 4.31E-04 | 12 |
| protein catabolic process | GO:0030163 | 4.33E-04 | 6 |
| regulation of kinase activity | GO:0043549 | 4.39E-04 | 6 |
| organic substance biosynthetic process | GO:1901576 | 4.61E-04 | 11 |
| cell projection organization | GO:0030030 | 4.70E-04 | 7 |
| regulation of multicellular organismal process | GO:0051239 | 4.71E-04 | 9 |
| anoikis | GO:0043276 | 4.94E-04 | 3 |
| regulation of gene expression | GO:0010468 | 5.22E-04 | 10 |
| biosynthetic process | GO:0009058 | 5.33E-04 | 11 |
| reproductive structure development | GO:0048608 | 6.25E-04 | 5 |
| regulation of mitotic cell cycle phase transition | GO:1901990 | 6.32E-04 | 5 |
| regulation of phosphorylation | GO:0042325 | 6.53E-04 | 7 |
| reproductive system development | GO:0061458 | 6.54E-04 | 5 |
| positive regulation of response to stimulus | GO:0048584 | 6.92E-04 | 8 |
| ameboidal-type cell migration | GO:0001667 | 7.25E-04 | 5 |
| cell morphogenesis | GO:0000902 | 7.30E-04 | 6 |
| positive regulation of epithelial cell migration | GO:0010634 | 7.67E-04 | 4 |
| positive regulation of protein kinase B signaling | GO:0051897 | 8.22E-04 | 4 |
| tube morphogenesis | GO:0035239 | 8.44E-04 | 6 |
| central nervous system development | GO:0007417 | 8.78E-04 | 6 |
| positive regulation of protein phosphorylation | GO:0001934 | 8.78E-04 | 6 |
| organ growth | GO:0035265 | 8.80E-04 | 4 |
| regulation of G1/S transition of mitotic cell cycle | GO:2000045 | 9.00E-04 | 4 |
| system development | GO:0048731 | 9.03E-04 | 10 |
| regulation of cell cycle phase transition | GO:1901987 | 9.42E-04 | 5 |
| positive regulation of signal transduction | GO:0009967 | 9.51E-04 | 7 |
| macromolecule biosynthetic process | GO:0009059 | 9.70E-04 | 10 |
| microtubule cytoskeleton organization | GO:0000226 | 9.72E-04 | 5 |
| endocrine pancreas development | GO:0031018 | 0.00102 | 3 |
| regulation of protein serine/threonine kinase activity | GO:0071900 | 0.00108 | 5 |
| regulation of cellular protein localization | GO:1903827 | 0.00117 | 5 |
| positive regulation of phosphorylation | GO:0042327 | 0.00119 | 6 |
| response to drug | GO:0042493 | 0.0012 | 6 |
| cellular component morphogenesis | GO:0032989 | 0.00126 | 6 |
| cellular response to inorganic substance | GO:0071241 | 0.00127 | 4 |
| positive regulation of protein catabolic process | GO:0045732 | 0.00127 | 4 |
| cellular component disassembly | GO:0022411 | 0.00131 | 5 |
| regulation of cell cycle G1/S phase transition | GO:1902806 | 0.00132 | 4 |
| positive regulation of intracellular signal transduction | GO:1902533 | 0.00132 | 6 |
| cellular response to acid chemical | GO:0071229 | 0.00141 | 4 |
| regulation of proteolysis involved in cellular protein catabolic process | GO:1903050 | 0.00146 | 4 |
| establishment of localization | GO:0051234 | 0.00147 | 10 |
| regulation of cell proliferation | GO:0042127 | 0.00149 | 7 |
| regulation of cellular macromolecule biosynthetic process | GO:2000112 | 0.00149 | 9 |
| ubiquitin-dependent protein catabolic process | GO:0006511 | 0.00154 | 5 |
| anatomical structure morphogenesis | GO:0009653 | 0.00155 | 8 |
| positive regulation of cellular component biogenesis | GO:0044089 | 0.00157 | 5 |
| modification-dependent protein catabolic process | GO:0019941 | 0.0016 | 5 |
| positive regulation of cell-matrix adhesion | GO:0001954 | 0.00171 | 3 |
| modification-dependent macromolecule catabolic process | GO:0043632 | 0.00171 | 5 |
| positive regulation of phosphate metabolic process | GO:0045937 | 0.0018 | 6 |
| positive regulation of phosphorus metabolic process | GO:0010562 | 0.0018 | 6 |
| extrinsic apoptotic signaling pathway | GO:0097191 | 0.00181 | 4 |
| protein localization to nucleus | GO:0034504 | 0.00181 | 4 |
| homeostatic process | GO:0042592 | 0.00184 | 7 |
| negative regulation of cellular protein metabolic process | GO:0032269 | 0.00212 | 6 |
| positive regulation of multicellular organismal process | GO:0051240 | 0.00213 | 7 |
| negative regulation of intracellular signal transduction | GO:1902532 | 0.00214 | 5 |
| chemotaxis | GO:0006935 | 0.00219 | 5 |
| positive regulation of DNA metabolic process | GO:0051054 | 0.00221 | 4 |
| taxis | GO:0042330 | 0.00223 | 5 |
| regulation of endothelial cell migration | GO:0010594 | 0.00242 | 4 |
| mitotic cell cycle phase transition | GO:0044772 | 0.00246 | 5 |
| tube development | GO:0035295 | 0.00262 | 6 |
| regulation of cellular protein catabolic process | GO:1903362 | 0.00268 | 4 |
| positive regulation of gene expression | GO:0010628 | 0.0027 | 7 |
| gene expression | GO:0010467 | 0.0027 | 10 |
| multicellular organism development | GO:0007275 | 0.00271 | 10 |
| negative regulation of molecular function | GO:0044092 | 0.00273 | 6 |
| regulation of cell-substrate junction assembly | GO:0090109 | 0.00296 | 3 |
| regulation of focal adhesion assembly | GO:0051893 | 0.00296 | 3 |
| positive regulation of protein modification process | GO:0031401 | 0.00299 | 6 |
| organonitrogen compound catabolic process | GO:1901565 | 0.00315 | 6 |
| regulation of cellular biosynthetic process | GO:0031326 | 0.00322 | 9 |
| proteolysis involved in cellular protein catabolic process | GO:0051603 | 0.00322 | 5 |
| cytoskeleton organization | GO:0007010 | 0.00324 | 6 |
| positive regulation of protein complex assembly | GO:0031334 | 0.00327 | 4 |
| cell cycle phase transition | GO:0044770 | 0.00353 | 5 |
| regulation of cell cycle | GO:0051726 | 0.00354 | 6 |
| positive regulation of transferase activity | GO:0051347 | 0.0037 | 5 |
| positive regulation of protein localization to nucleus | GO:1900182 | 0.00375 | 3 |
| regulation of hydrolase activity | GO:0051336 | 0.00379 | 6 |
| developmental growth | GO:0048589 | 0.00401 | 5 |
| positive regulation of cellular component organization | GO:0051130 | 0.00405 | 6 |
| regulation of adherens junction organization | GO:1903391 | 0.00411 | 3 |
| regulation of mitotic cell cycle | GO:0007346 | 0.00413 | 5 |
| response to UV-A | GO:0070141 | 0.00414 | 2 |
| organic substance catabolic process | GO:1901575 | 0.00415 | 7 |
| microtubule-based process | GO:0007017 | 0.00433 | 5 |
| cell development | GO:0048468 | 0.00456 | 7 |
| positive regulation of proteasomal ubiquitin-dependent protein catabolic process | GO:0032436 | 0.00468 | 3 |
| G1/S transition of mitotic cell cycle | GO:0000082 | 0.00473 | 4 |
| cellular response to hormone stimulus | GO:0032870 | 0.00475 | 5 |
| developmental process involved in reproduction | GO:0003006 | 0.00478 | 5 |
| regulation of mitochondrial membrane potential | GO:0051881 | 0.00489 | 3 |
| endothelial cell migration | GO:0043542 | 0.00494 | 4 |
| cellular protein catabolic process | GO:0044257 | 0.00516 | 5 |
| macromolecule catabolic process | GO:0009057 | 0.00542 | 6 |
| pancreas development | GO:0031016 | 0.00553 | 3 |
| regulation of oxidative stress-induced cell death | GO:1903201 | 0.00553 | 3 |
| anatomical structure development | GO:0048856 | 0.00569 | 10 |
| cellular response to growth factor stimulus | GO:0071363 | 0.00589 | 5 |
| cellular biosynthetic process | GO:0044249 | 0.00591 | 10 |
| cell cycle G1/S phase transition | GO:0044843 | 0.0062 | 4 |
| negative regulation of signal transduction | GO:0009968 | 0.00625 | 6 |
| regulation of multicellular organismal development | GO:2000026 | 0.00649 | 7 |
| regulation of MAPK cascade | GO:0043408 | 0.00655 | 5 |
| immune system process | GO:0002376 | 0.00672 | 8 |
| cellular response to peptide hormone stimulus | GO:0071375 | 0.00681 | 4 |
| negative regulation of catabolic process | GO:0009895 | 0.00681 | 4 |
| cell-substrate adherens junction assembly | GO:0007045 | 0.00698 | 3 |
| focal adhesion assembly | GO:0048041 | 0.00698 | 3 |
| positive regulation of phosphatidylinositol 3-kinase signaling | GO:0014068 | 0.00698 | 3 |
| response to growth factor | GO:0070848 | 0.00728 | 5 |
| cellular response to endogenous stimulus | GO:0071495 | 0.00734 | 6 |
| negative regulation of autophagy | GO:0010507 | 0.00751 | 3 |
| signal complex assembly | GO:0007172 | 0.00773 | 2 |
| positive regulation of cell death | GO:0010942 | 0.0078 | 5 |
| negative regulation of mitotic cell cycle | GO:0045930 | 0.00797 | 4 |
| negative regulation of cellular macromolecule biosynthetic process | GO:2000113 | 0.00819 | 6 |
| regulation of cellular response to oxidative stress | GO:1900407 | 0.00896 | 3 |
| negative regulation of cell communication | GO:0010648 | 0.00955 | 6 |
| negative regulation of signaling | GO:0023057 | 0.00972 | 6 |
| cellular macromolecule biosynthetic process | GO:0034645 | 0.0098 | 9 |
| adherens junction assembly | GO:0034333 | 0.00992 | 3 |
| cellular response to ketone | GO:1901655 | 0.00992 | 3 |
